# Supplementary material for: Transcriptome Analyses Identify Potential Key microRNAs and Their Target Genes Contributing to Ovarian Reserve
Source: Int J Mol Sci. 2021 Oct 6;22(19):10819. doi: 10.3390/ijms221910819 (PMC8509654; doi:10.3390/ijms221910819)
Supplement: Supplementary file 1 [file ijms-22-10819-s001.zip › Supplementary Figures.pptx]

## Slide 1
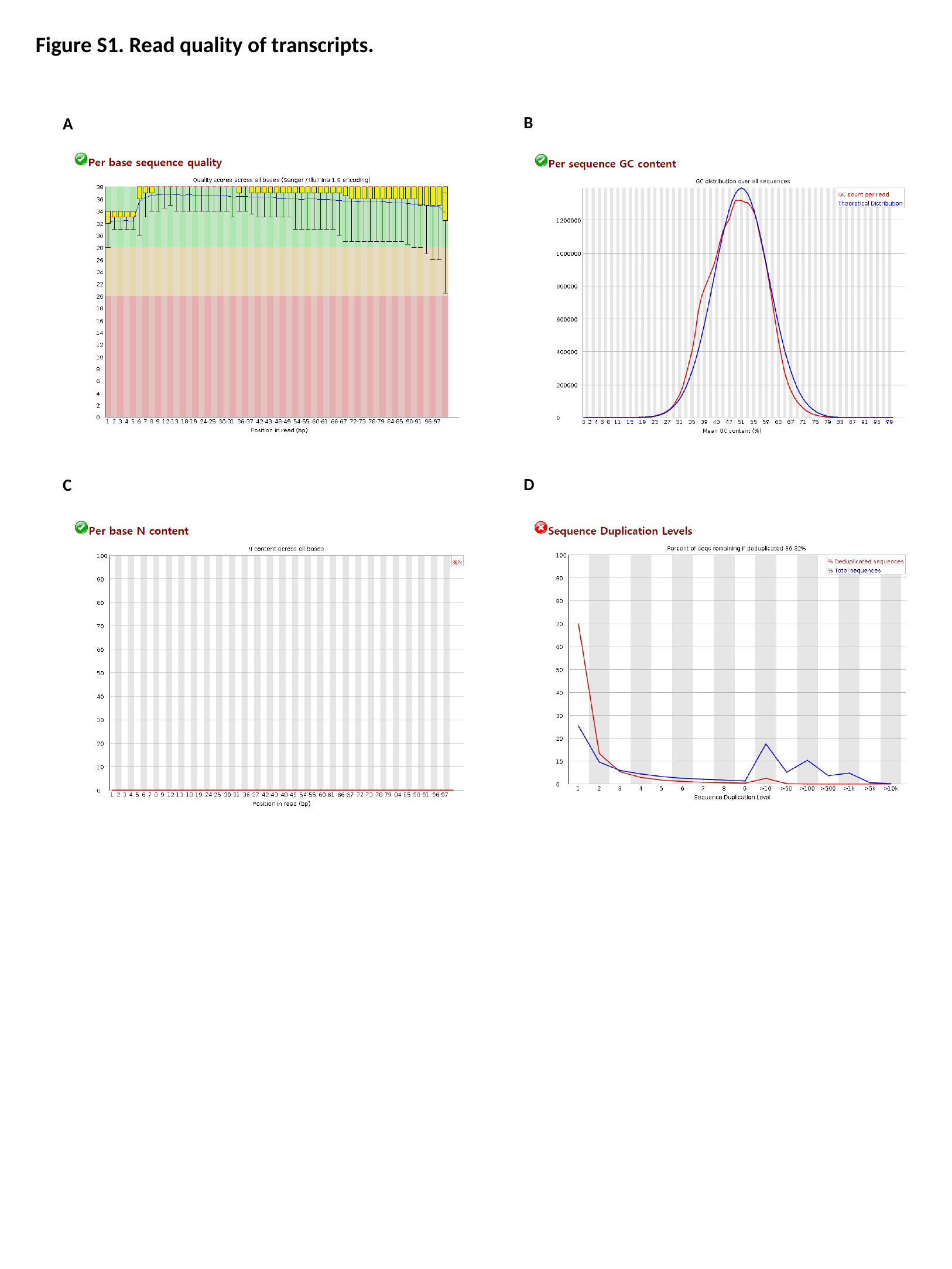

Figure S1. Read quality of transcripts.
B
A
D
C

## Slide 2
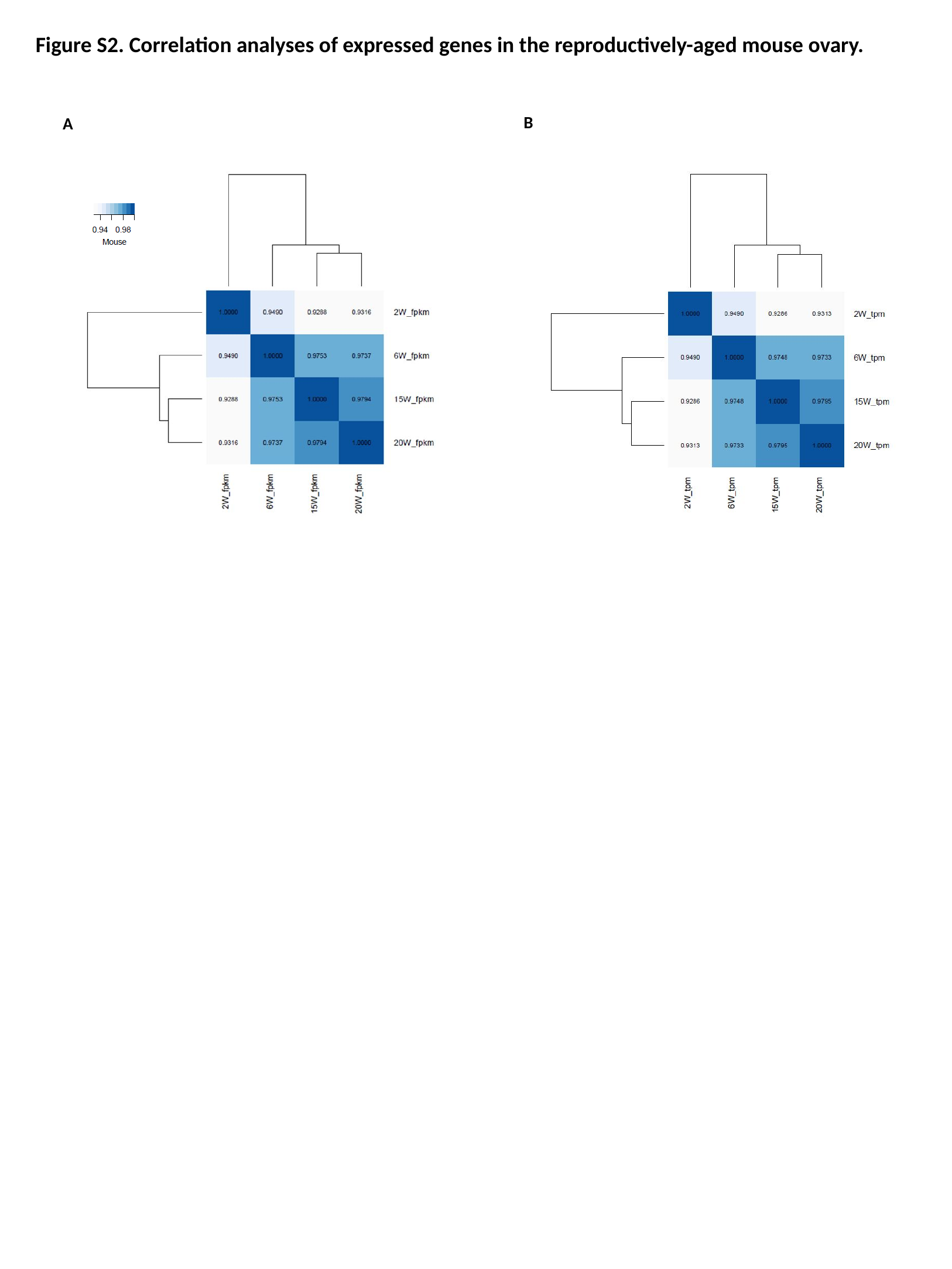

Figure S2. Correlation analyses of expressed genes in the reproductively-aged mouse ovary.
B
A

## Slide 3
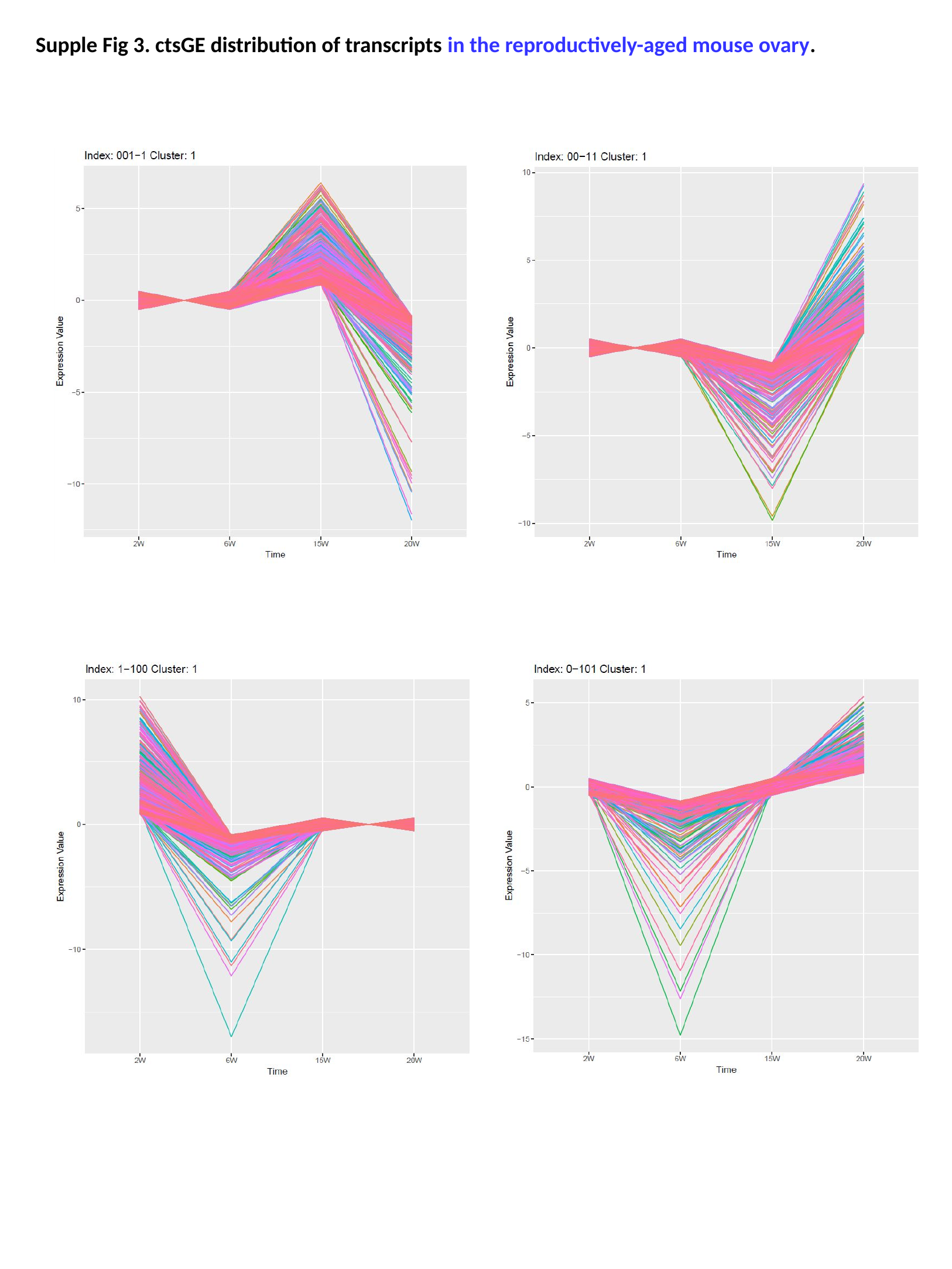

Supple Fig 3. ctsGE distribution of transcripts in the reproductively-aged mouse ovary.

## Slide 4
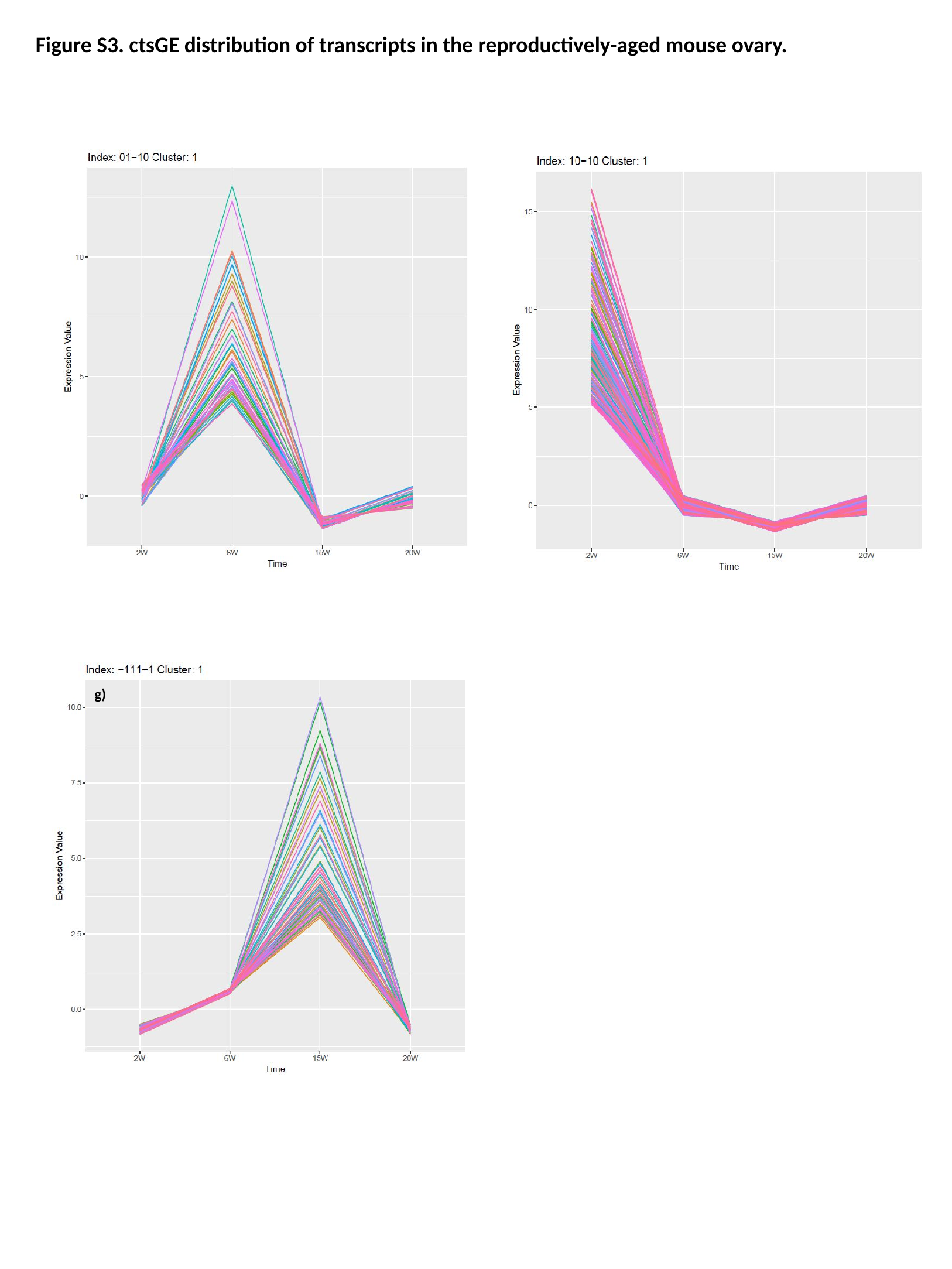

Figure S3. ctsGE distribution of transcripts in the reproductively-aged mouse ovary.
g)

## Slide 5
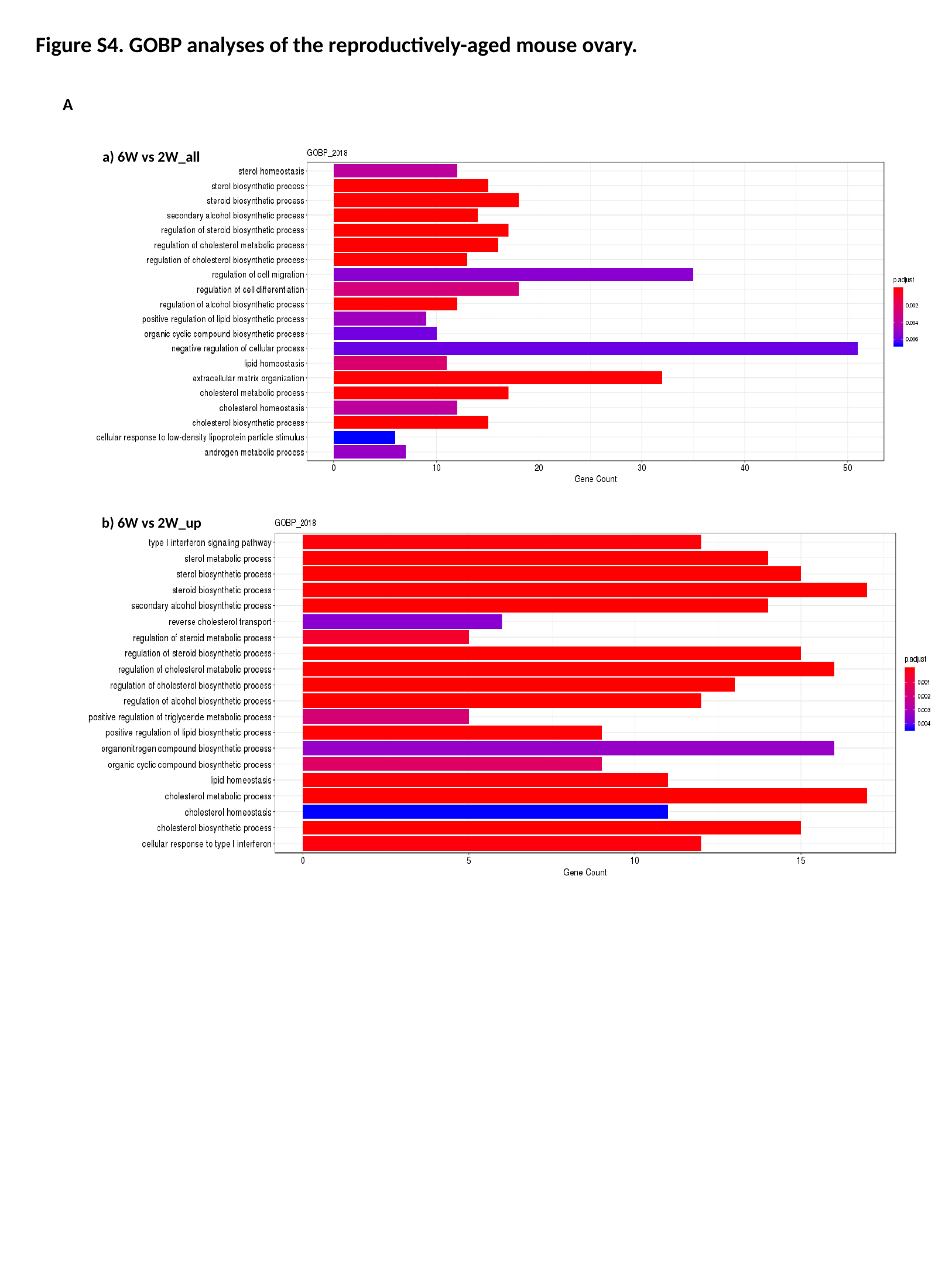

Figure S4. GOBP analyses of the reproductively-aged mouse ovary.
A
a) 6W vs 2W_all
b) 6W vs 2W_up

## Slide 6
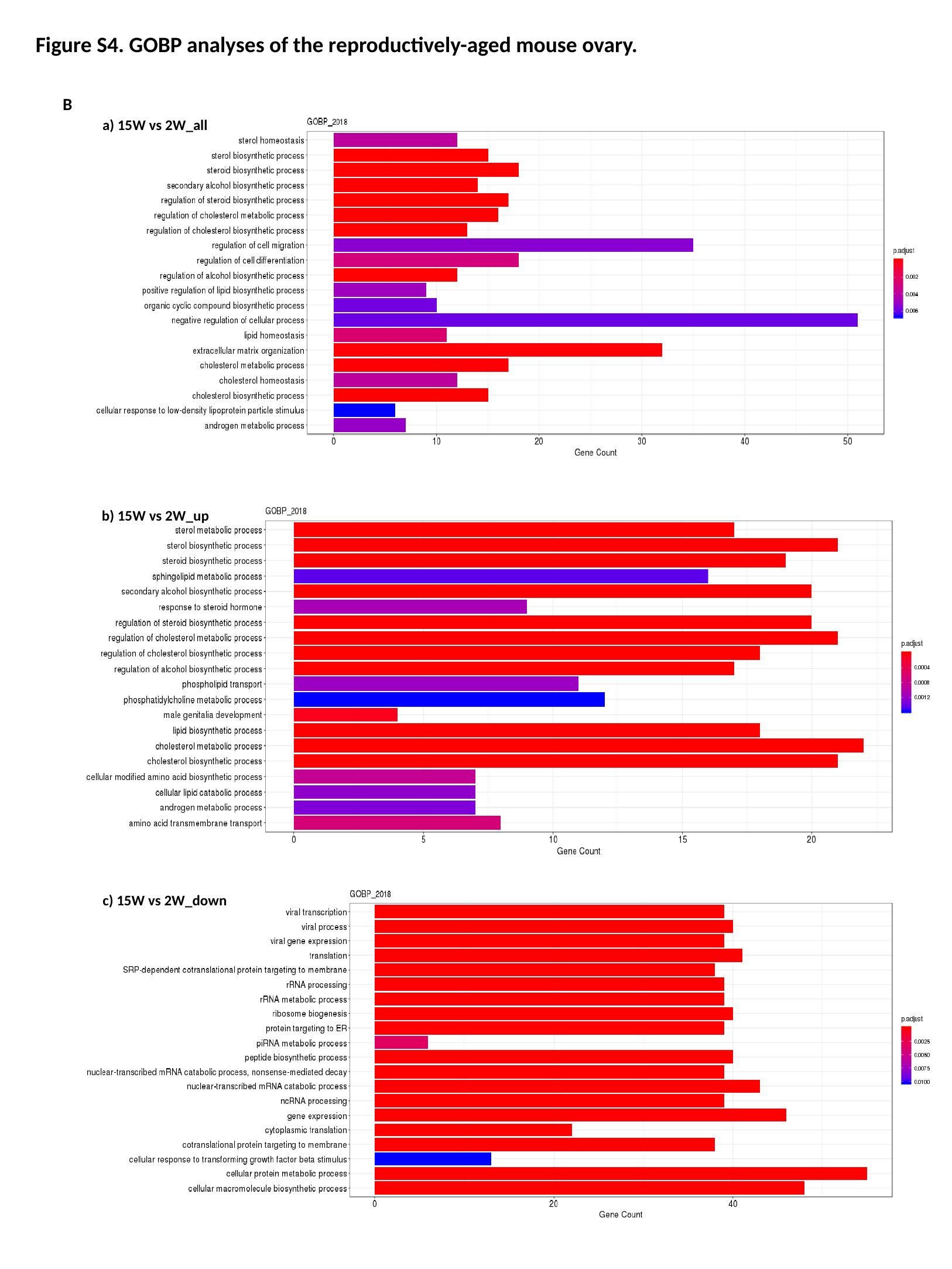

Figure S4. GOBP analyses of the reproductively-aged mouse ovary.
B
a) 15W vs 2W_all
b) 15W vs 2W_up
c) 15W vs 2W_down

## Slide 7
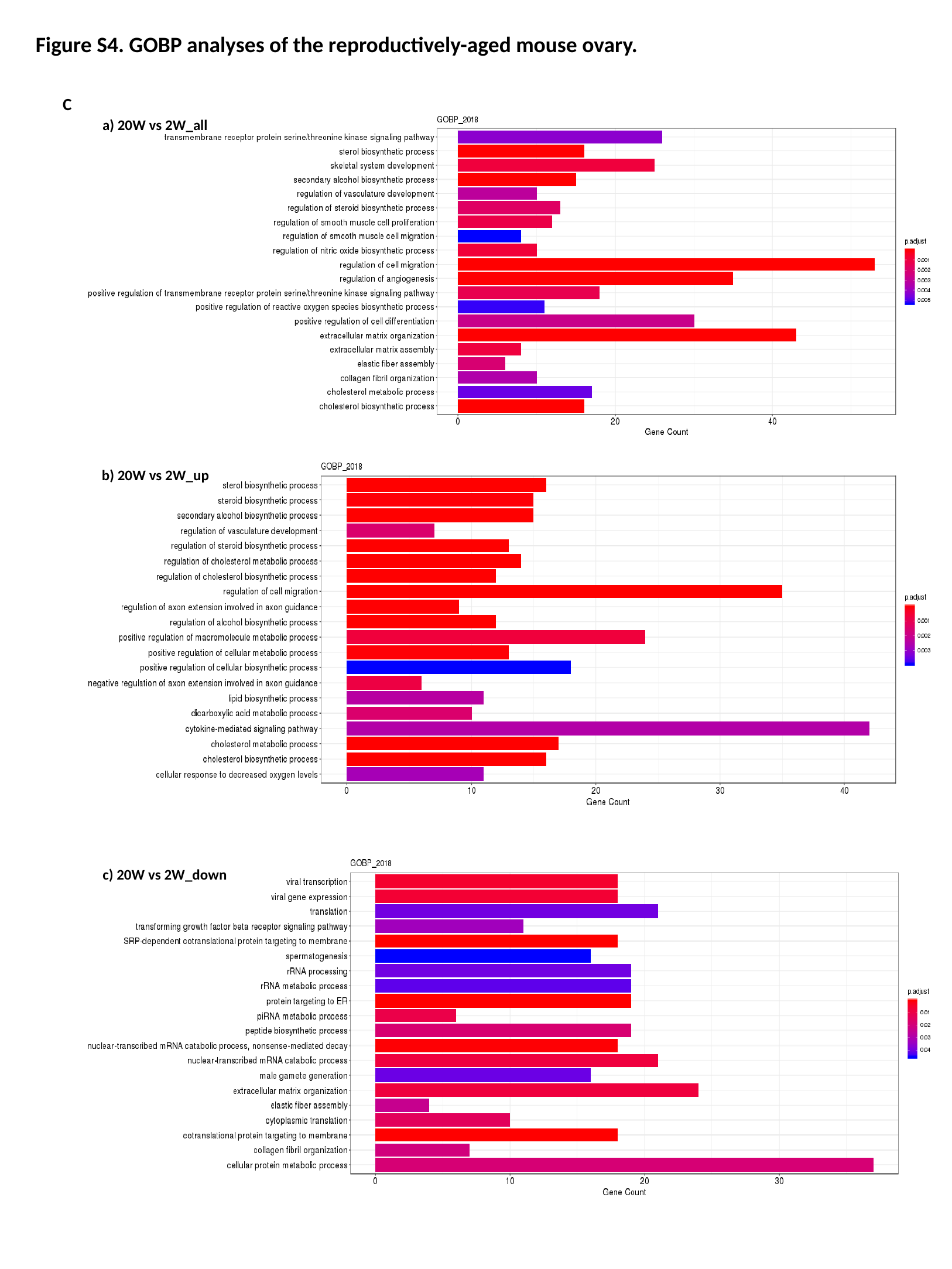

Figure S4. GOBP analyses of the reproductively-aged mouse ovary.
C
a) 20W vs 2W_all
b) 20W vs 2W_up
c) 20W vs 2W_down

## Slide 8
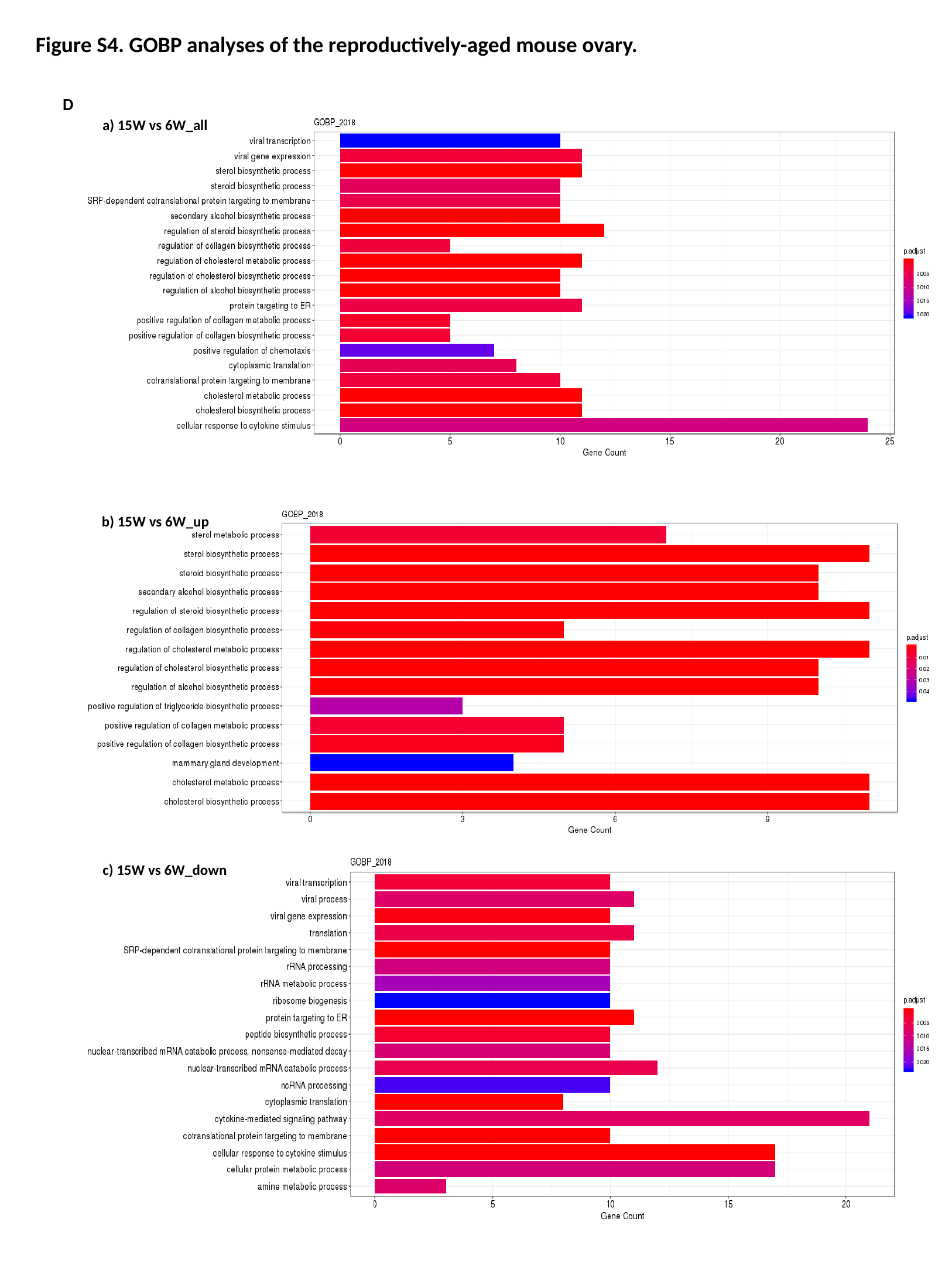

Figure S4. GOBP analyses of the reproductively-aged mouse ovary.
D
a) 15W vs 6W_all
b) 15W vs 6W_up
c) 15W vs 6W_down

## Slide 9
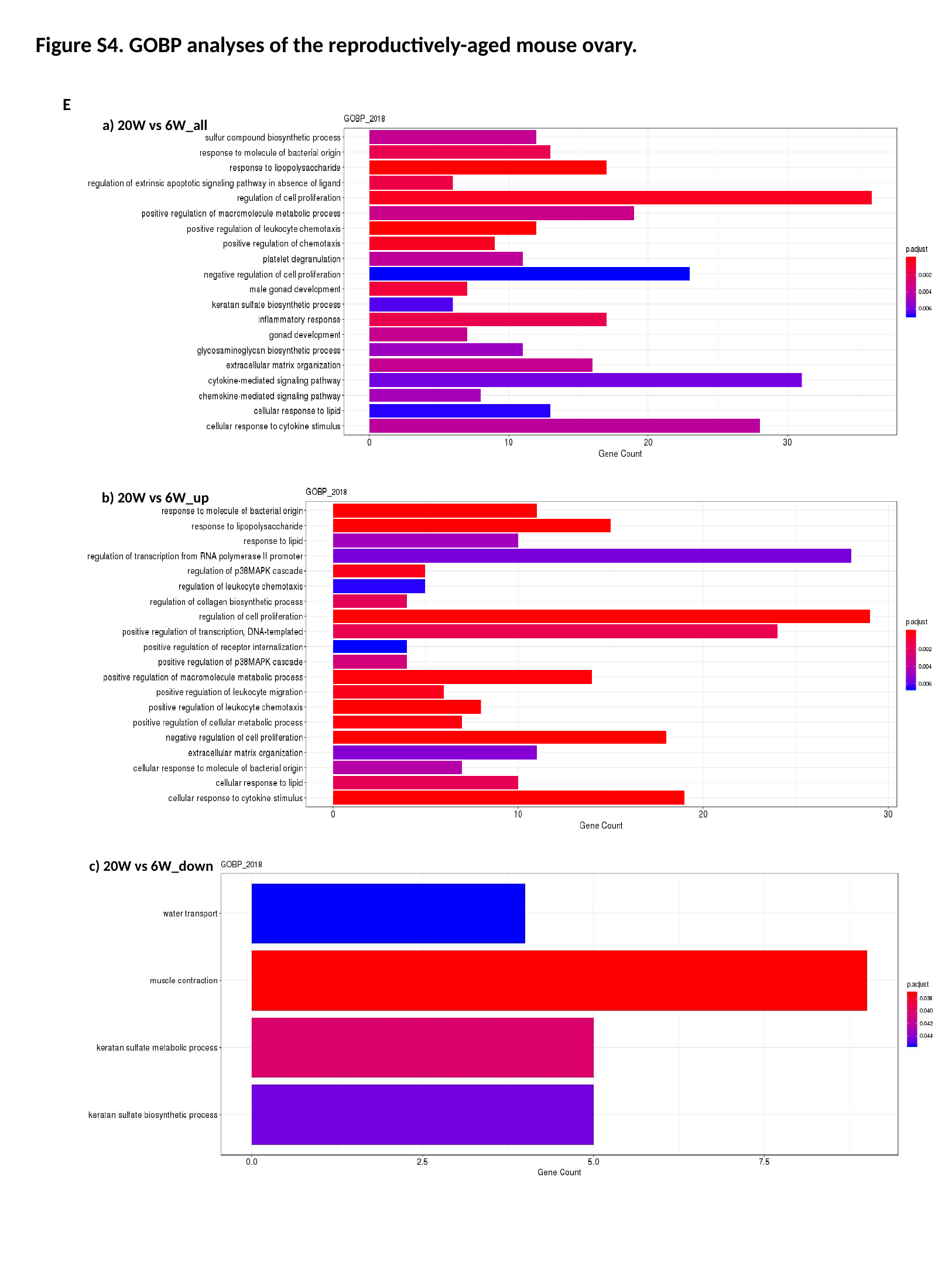

Figure S4. GOBP analyses of the reproductively-aged mouse ovary.
E
a) 20W vs 6W_all
b) 20W vs 6W_up
c) 20W vs 6W_down

## Slide 10
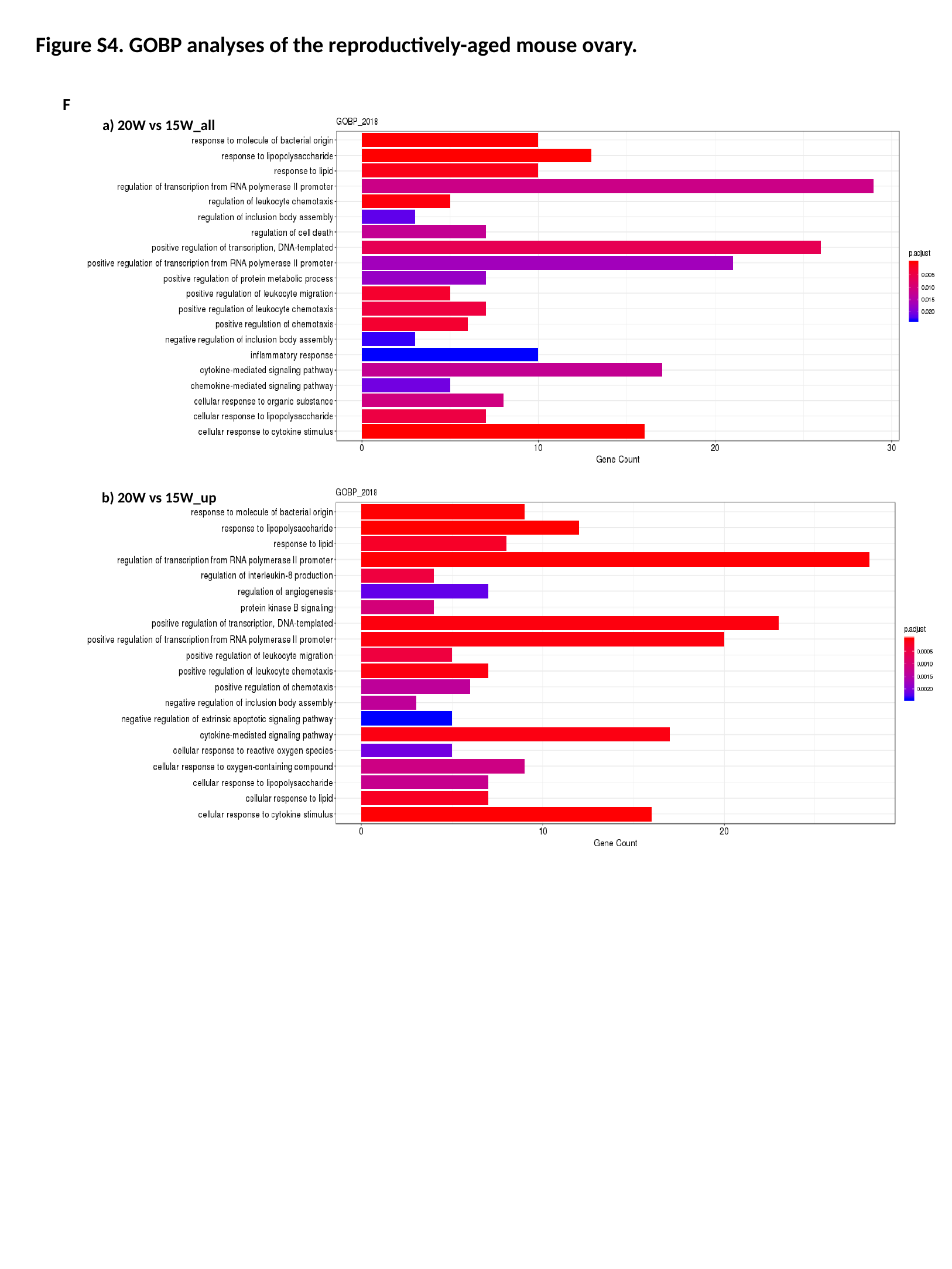

Figure S4. GOBP analyses of the reproductively-aged mouse ovary.
F
a) 20W vs 15W_all
b) 20W vs 15W_up

## Slide 11
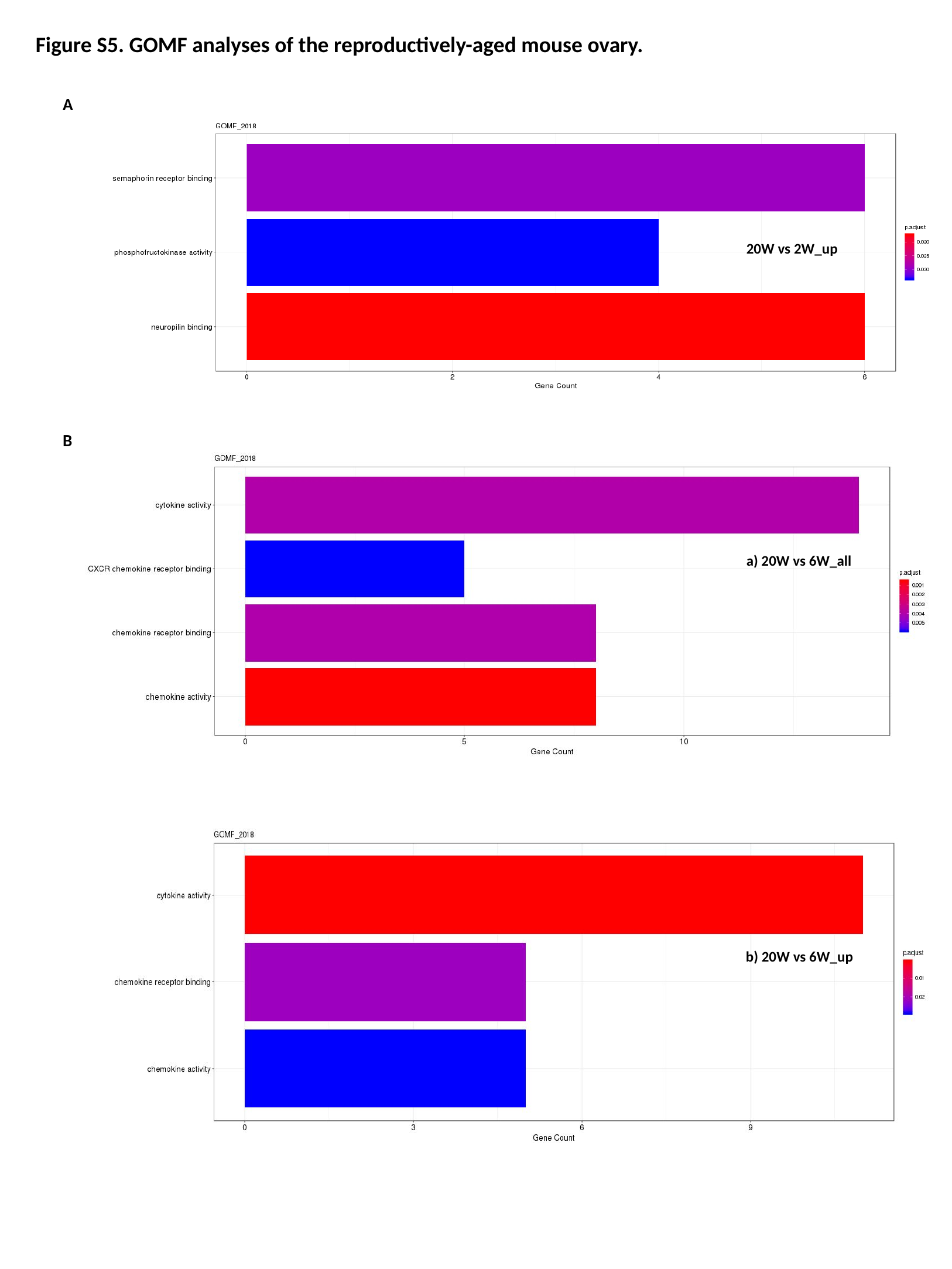

Figure S5. GOMF analyses of the reproductively-aged mouse ovary.
A
20W vs 2W_up
B
a) 20W vs 6W_all
b) 20W vs 6W_up

## Slide 12
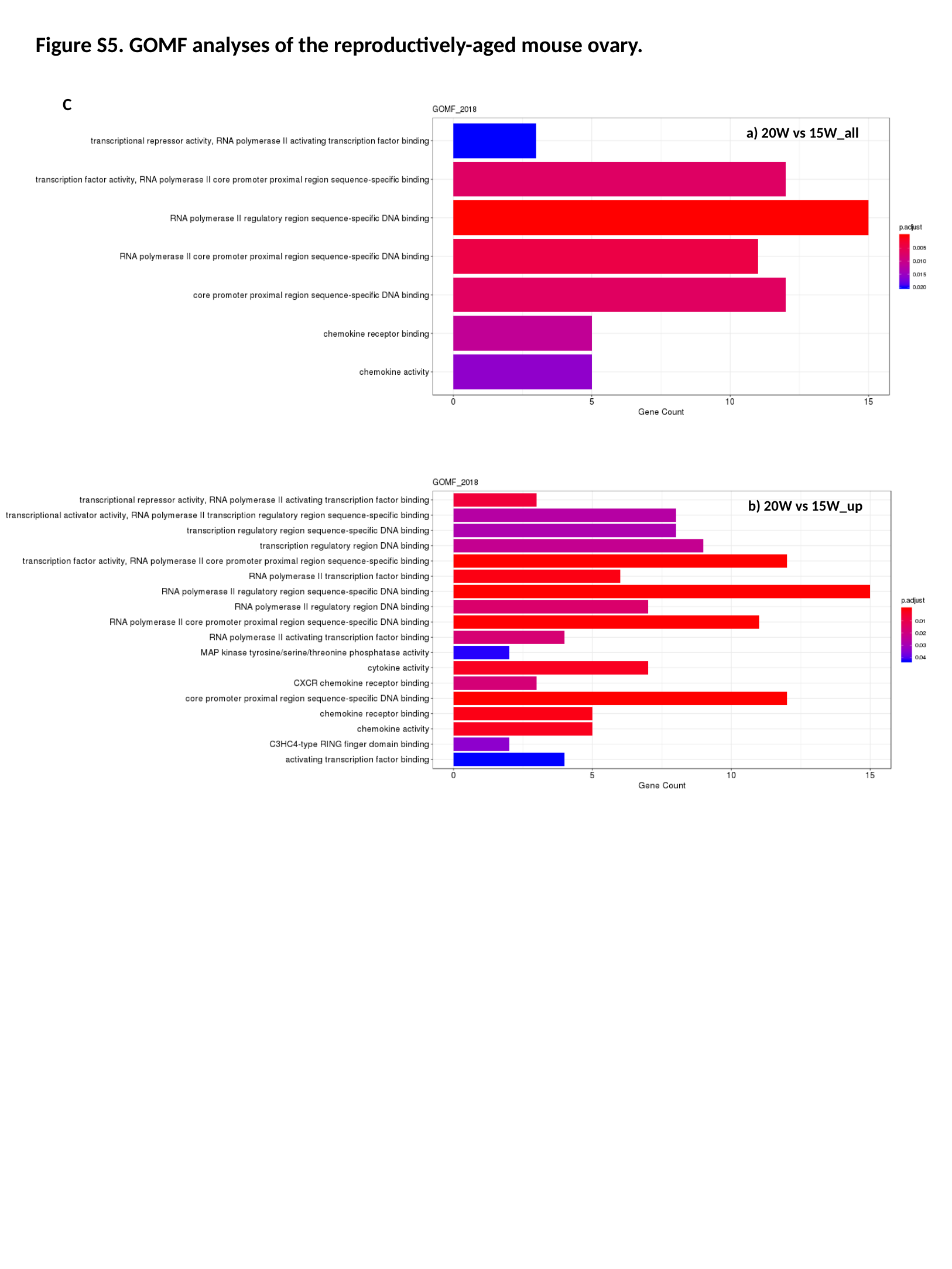

Figure S5. GOMF analyses of the reproductively-aged mouse ovary.
C
a) 20W vs 15W_all
b) 20W vs 15W_up

## Slide 13
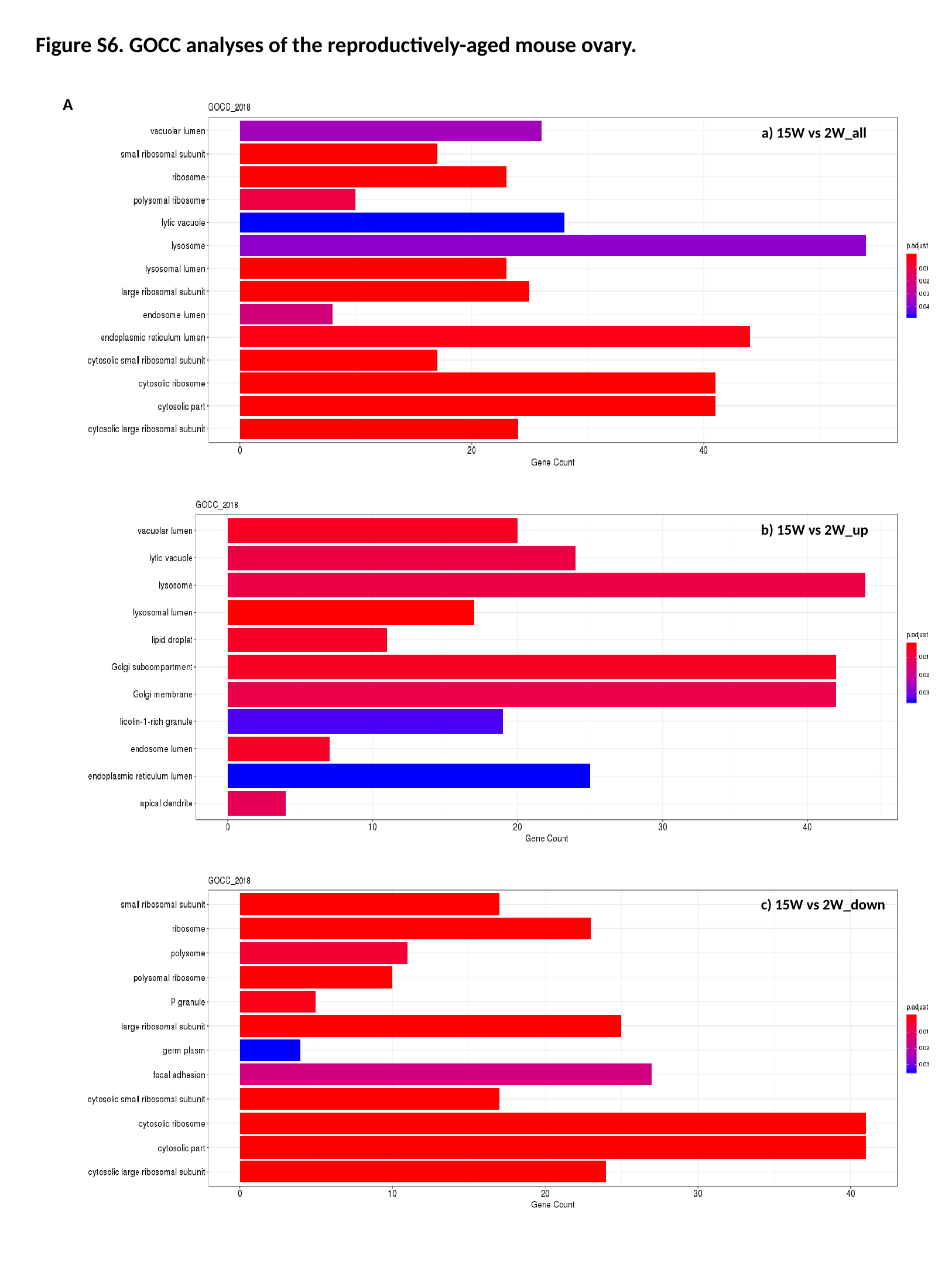

Figure S6. GOCC analyses of the reproductively-aged mouse ovary.
A
a) 15W vs 2W_all
b) 15W vs 2W_up
c) 15W vs 2W_down

## Slide 14
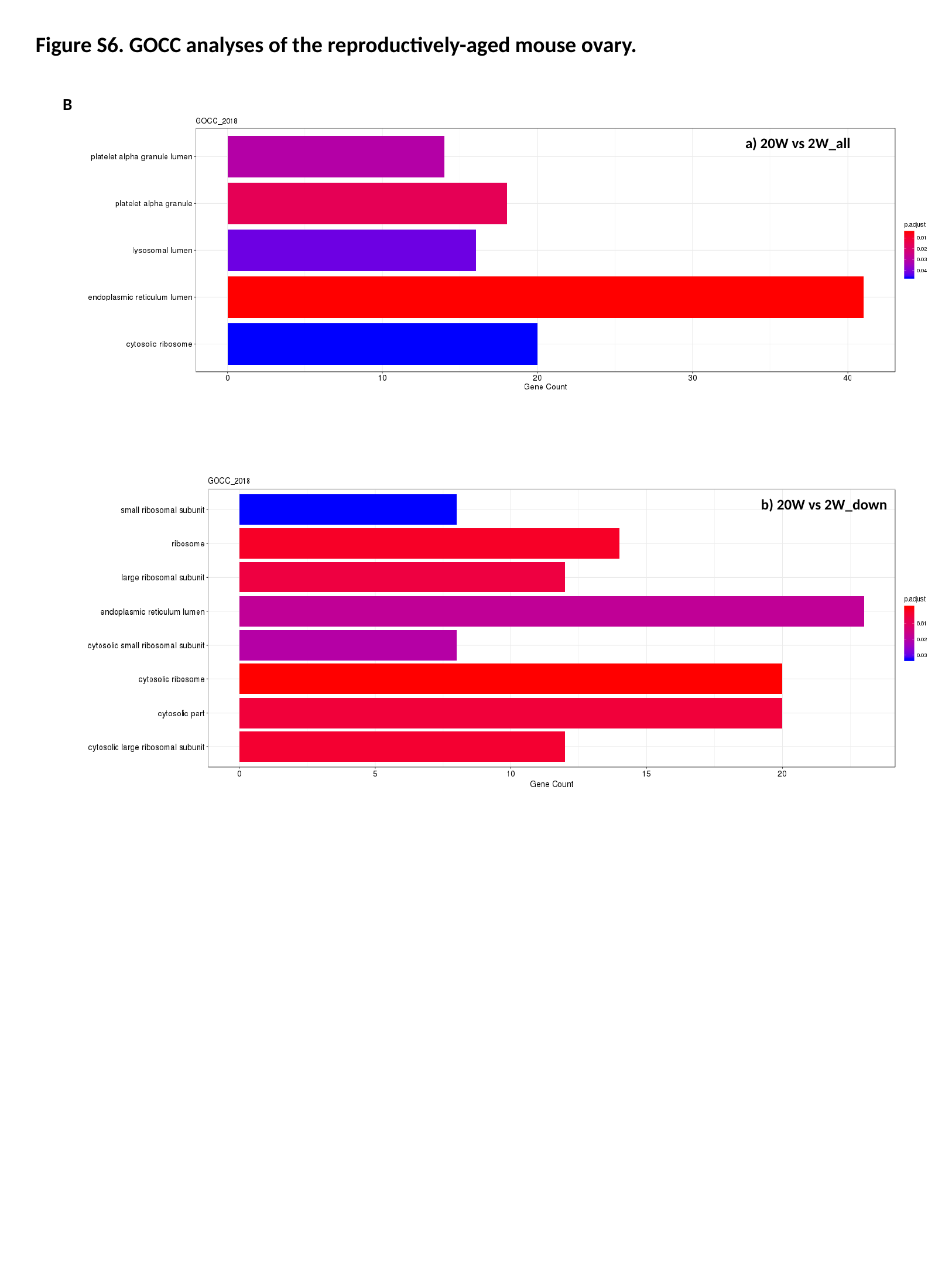

Figure S6. GOCC analyses of the reproductively-aged mouse ovary.
B
a) 20W vs 2W_all
b) 20W vs 2W_down

## Slide 15
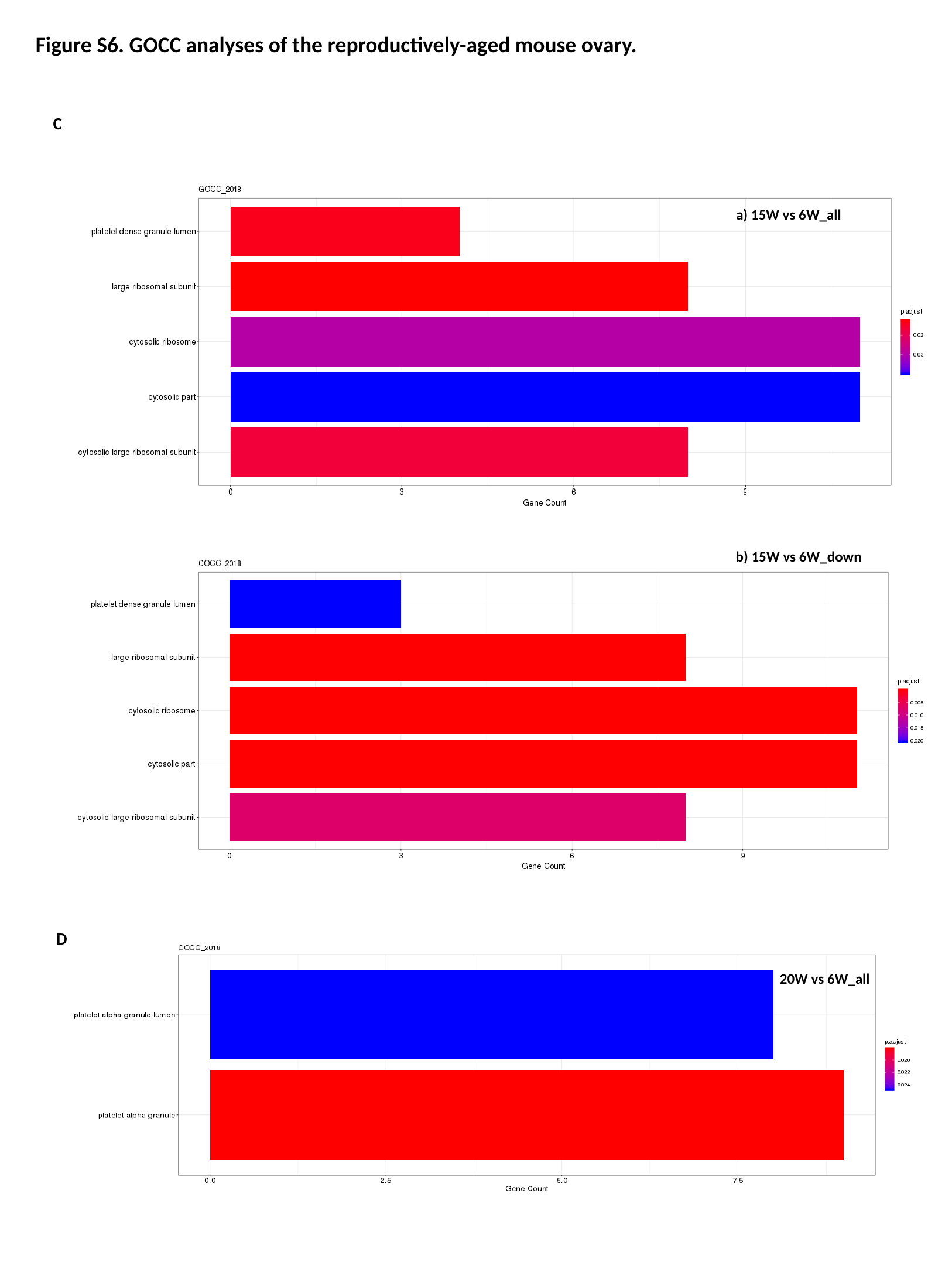

Figure S6. GOCC analyses of the reproductively-aged mouse ovary.
C
a) 15W vs 6W_all
b) 15W vs 6W_down
D
20W vs 6W_all

## Slide 16
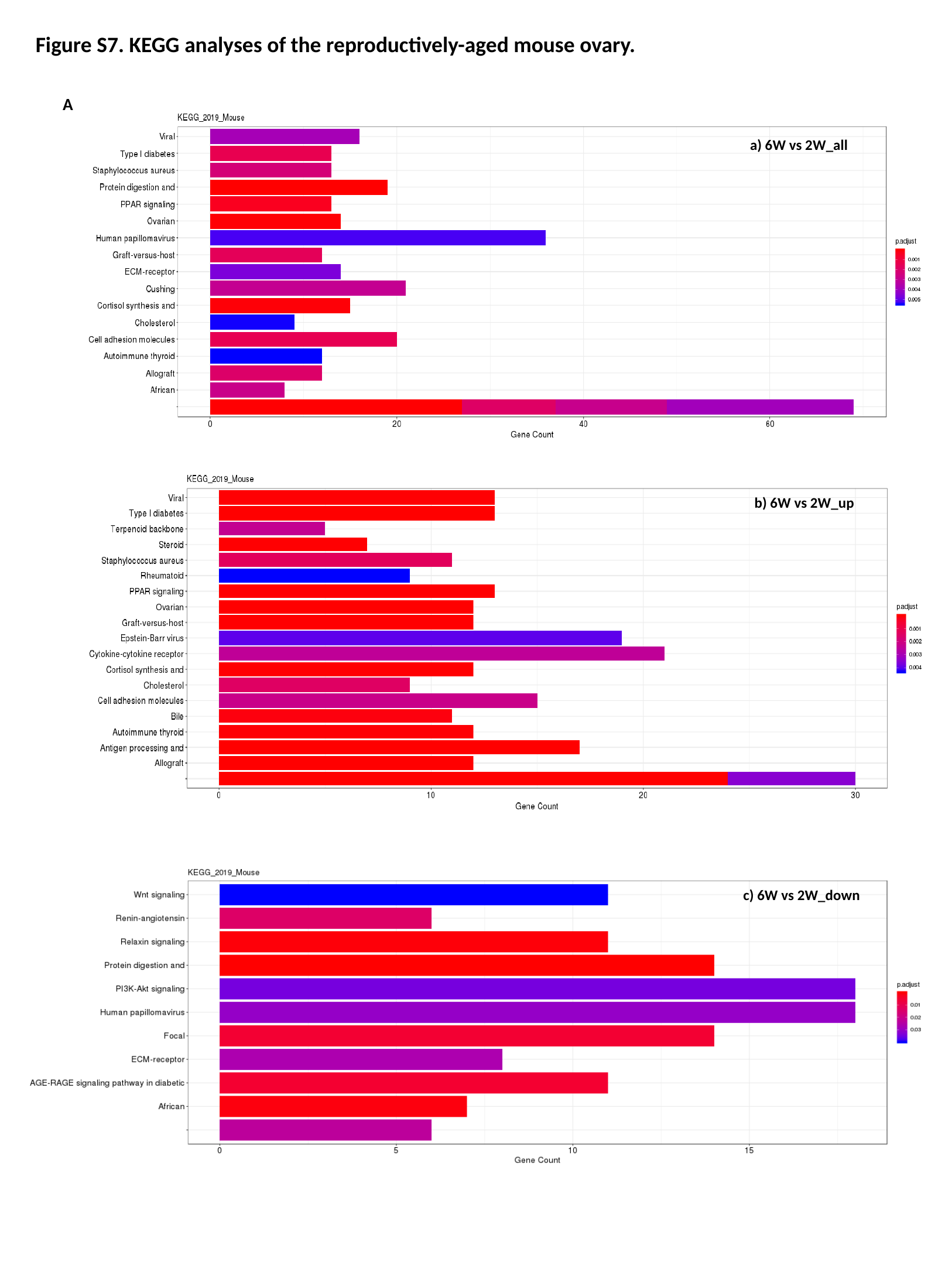

Figure S7. KEGG analyses of the reproductively-aged mouse ovary.
A
a) 6W vs 2W_all
b) 6W vs 2W_up
c) 6W vs 2W_down

## Slide 17
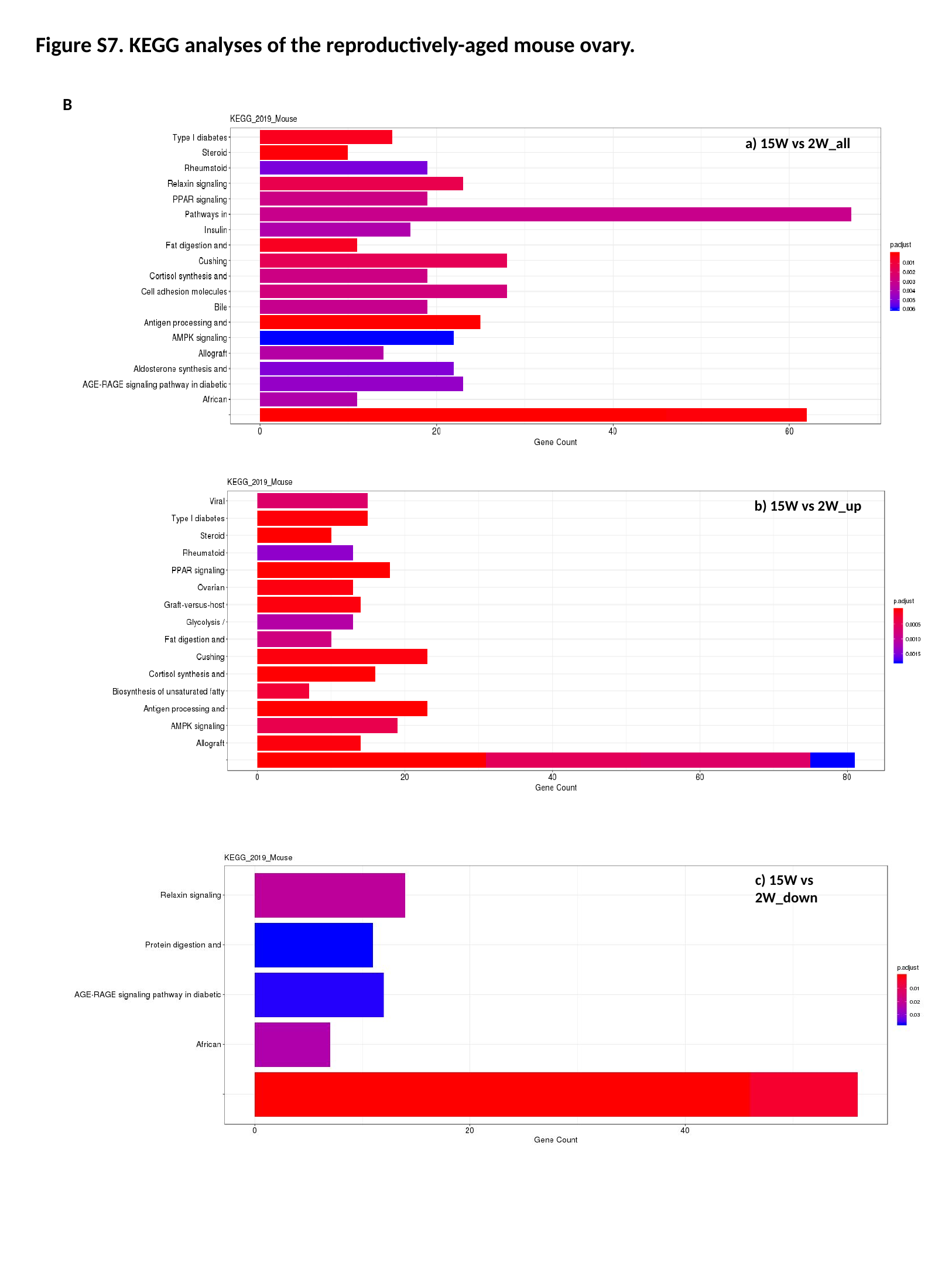

Figure S7. KEGG analyses of the reproductively-aged mouse ovary.
B
a) 15W vs 2W_all
b) 15W vs 2W_up
c) 15W vs 2W_down

## Slide 18
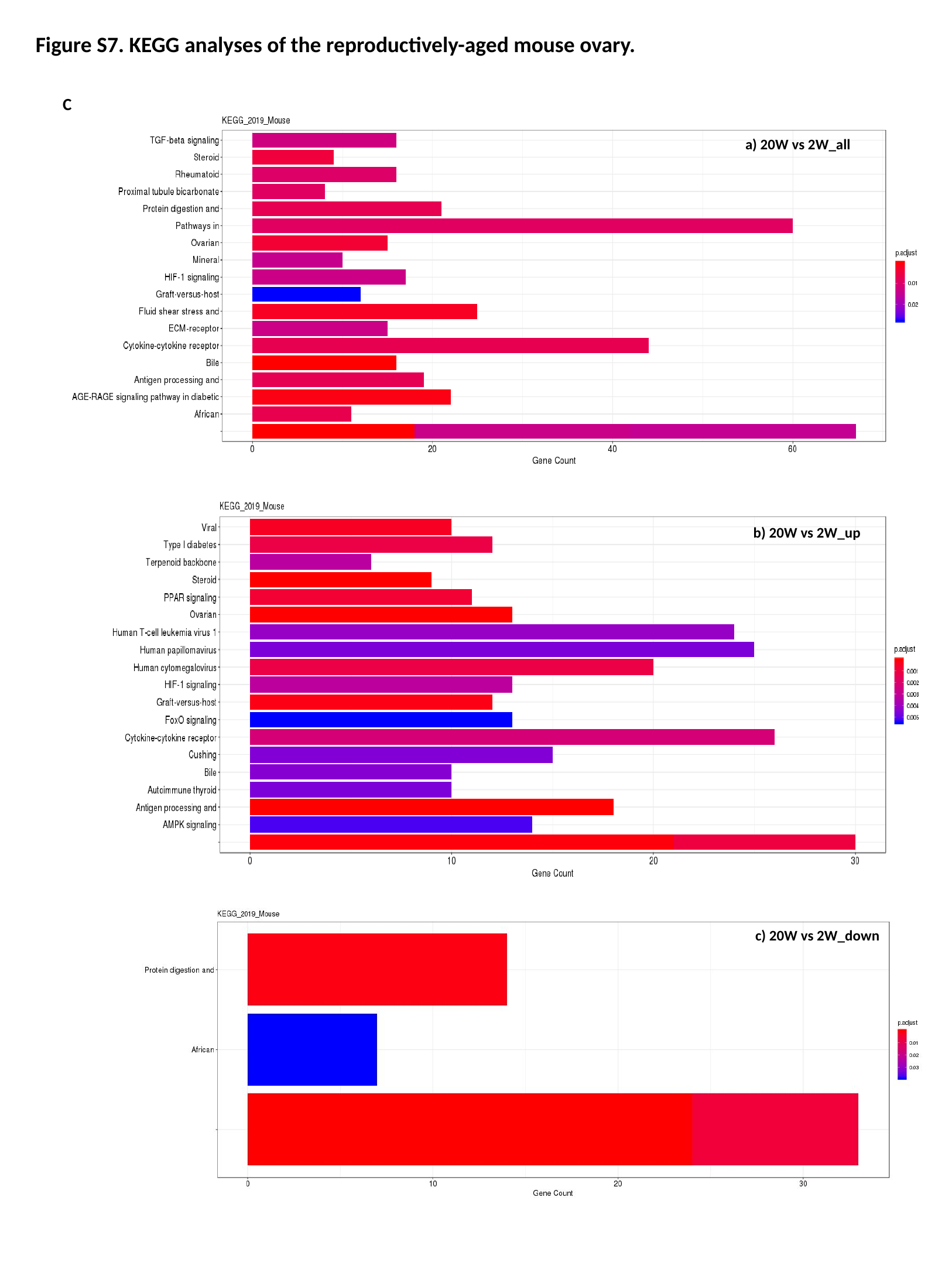

Figure S7. KEGG analyses of the reproductively-aged mouse ovary.
C
a) 20W vs 2W_all
b) 20W vs 2W_up
c) 20W vs 2W_down

## Slide 19
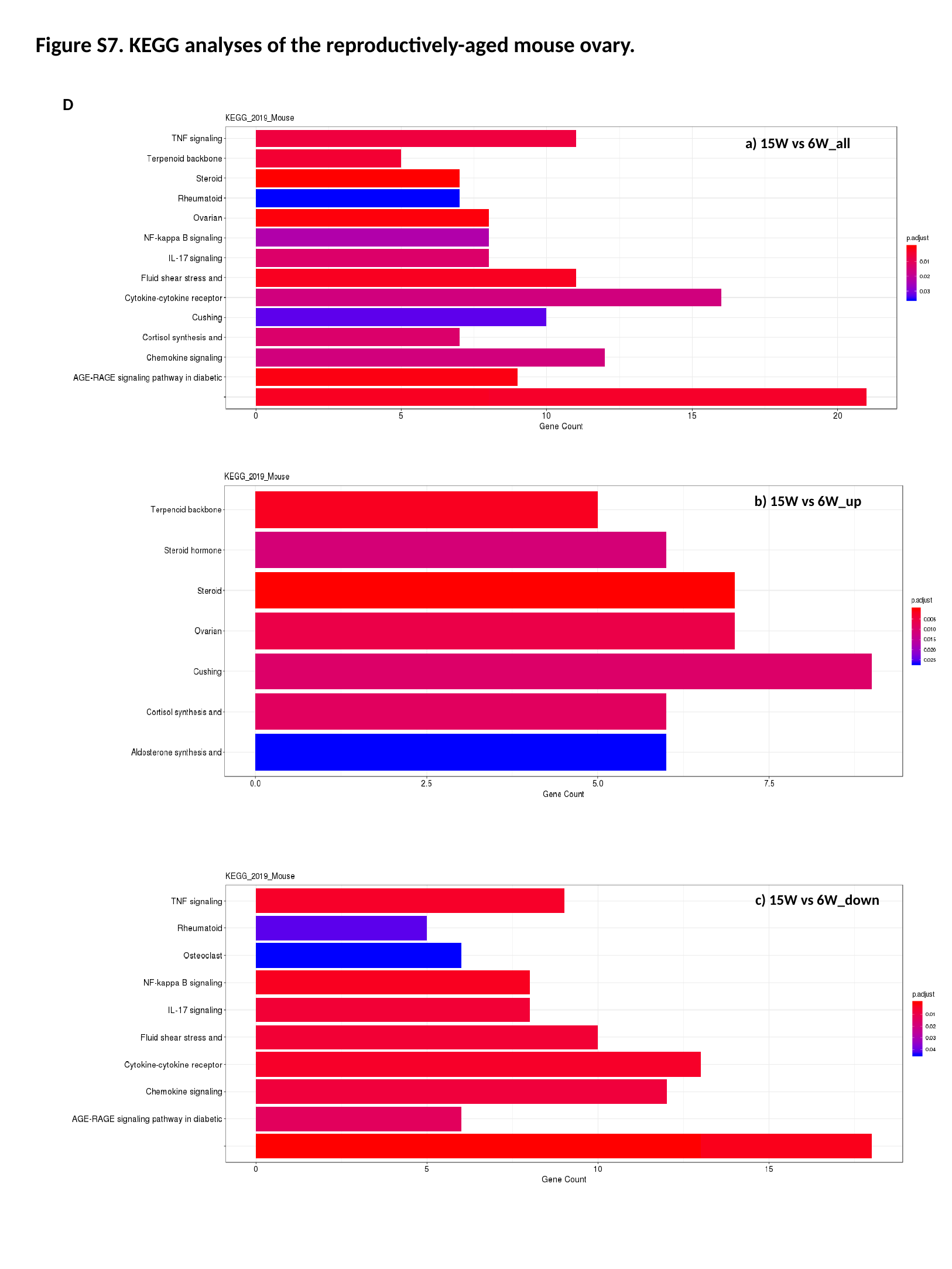

Figure S7. KEGG analyses of the reproductively-aged mouse ovary.
D
a) 15W vs 6W_all
b) 15W vs 6W_up
c) 15W vs 6W_down

## Slide 20
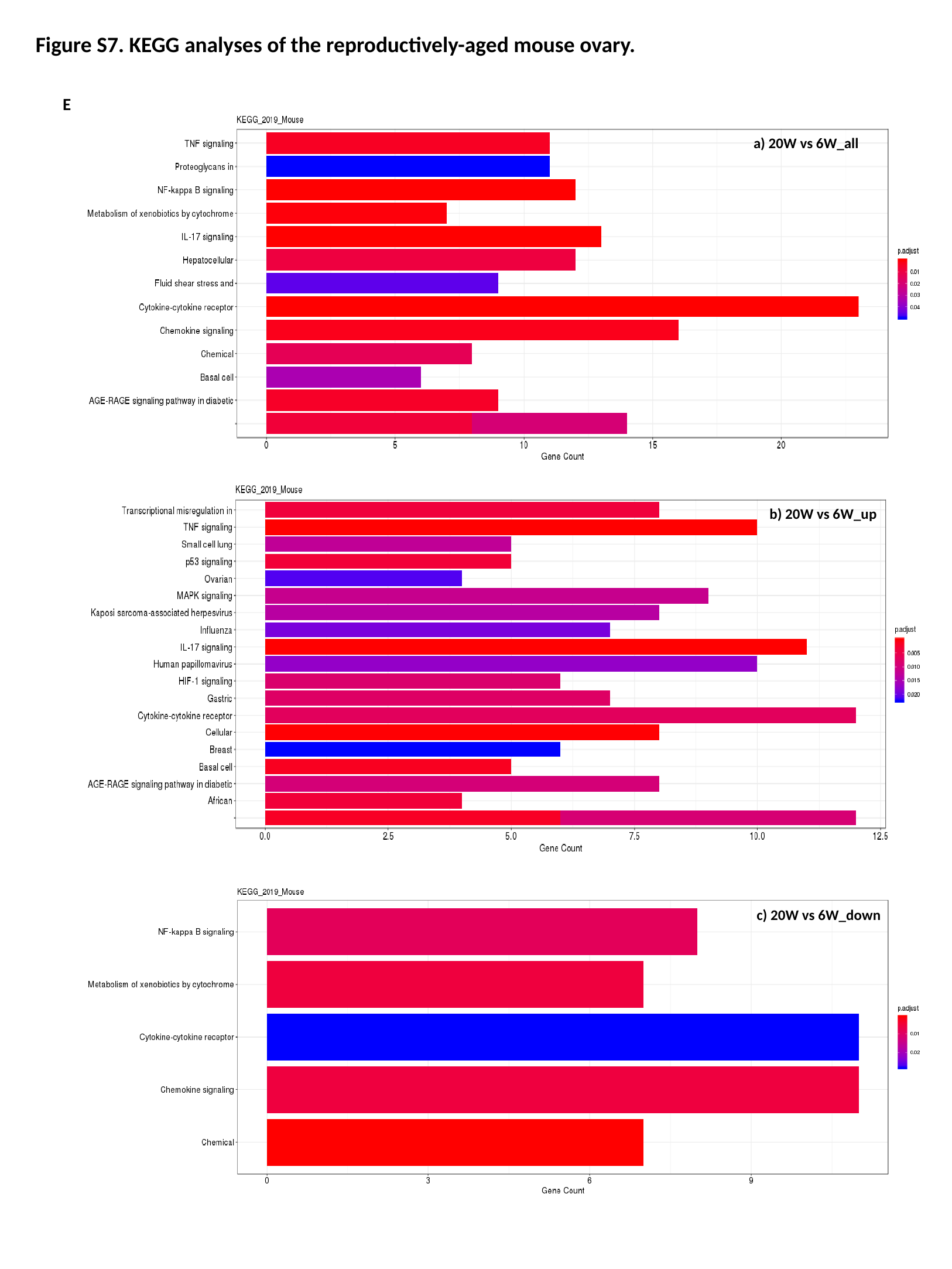

Figure S7. KEGG analyses of the reproductively-aged mouse ovary.
E
a) 20W vs 6W_all
b) 20W vs 6W_up
c) 20W vs 6W_down

## Slide 21
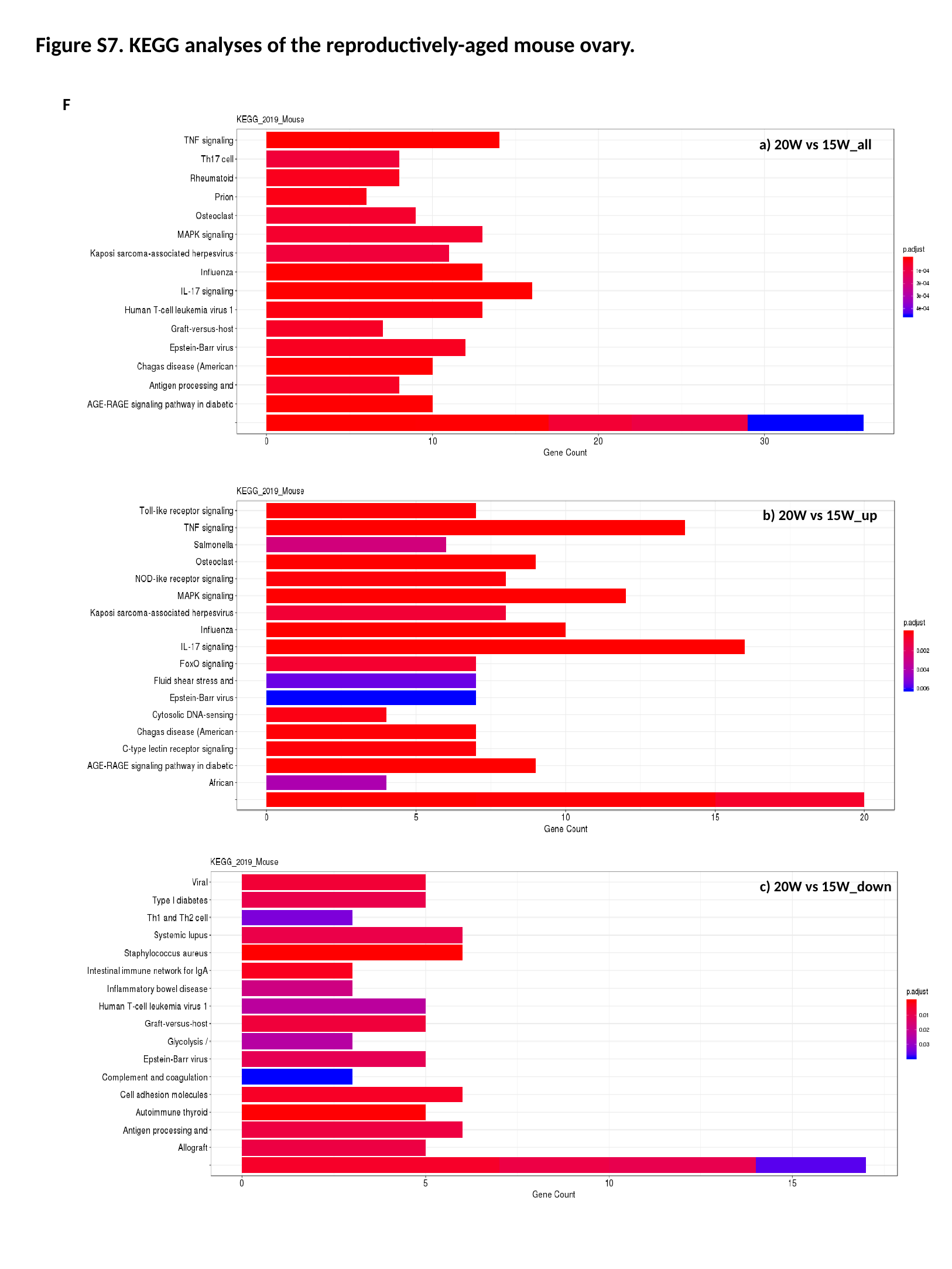

Figure S7. KEGG analyses of the reproductively-aged mouse ovary.
F
a) 20W vs 15W_all
b) 20W vs 15W_up
c) 20W vs 15W_down

## Slide 22
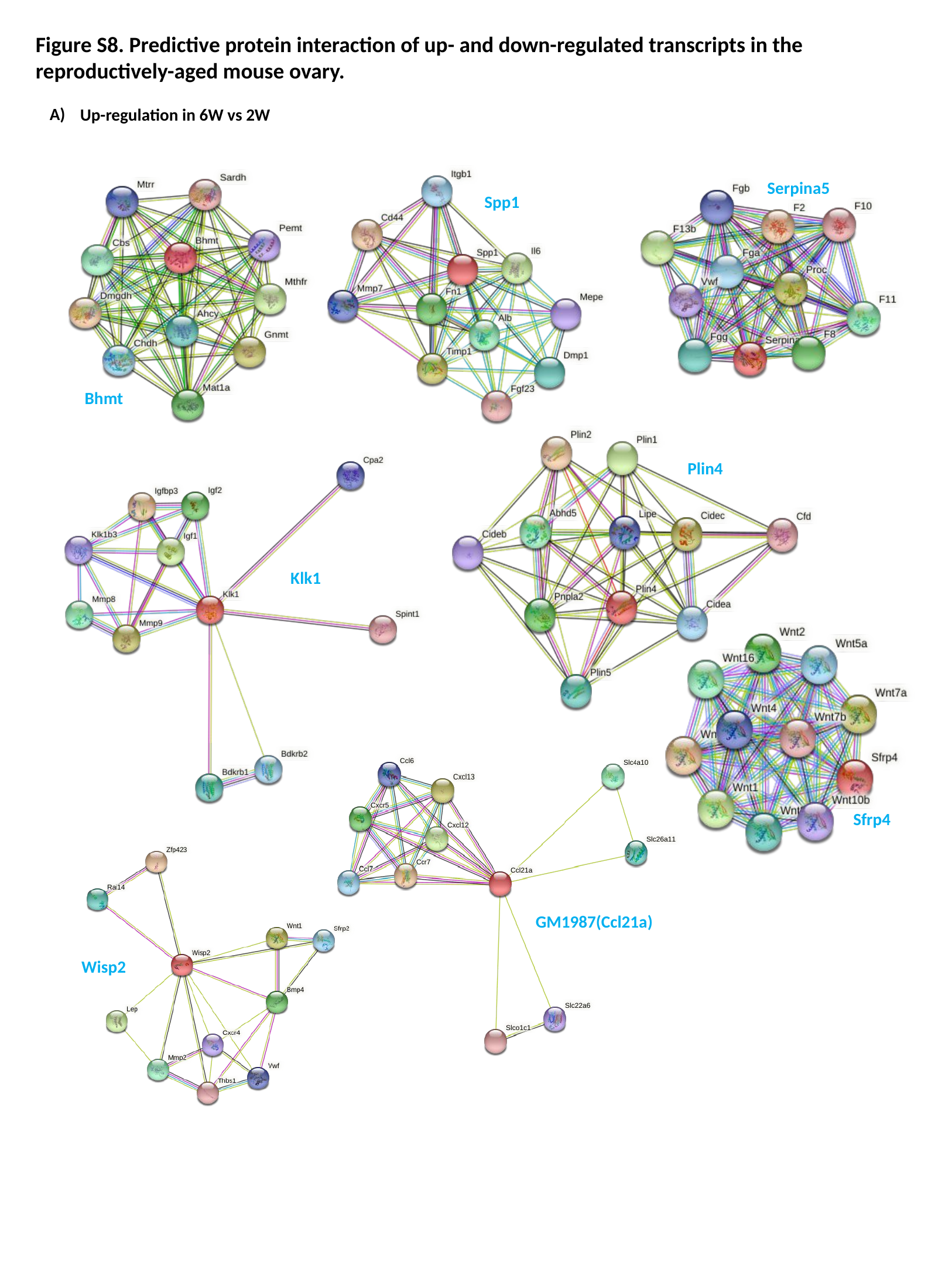

Figure S8. Predictive protein interaction of up- and down-regulated transcripts in the reproductively-aged mouse ovary.
A)
Up-regulation in 6W vs 2W
Spp1
Bhmt
Serpina5
Plin4
Klk1
Sfrp4
GM1987(Ccl21a)
Wisp2

## Slide 23
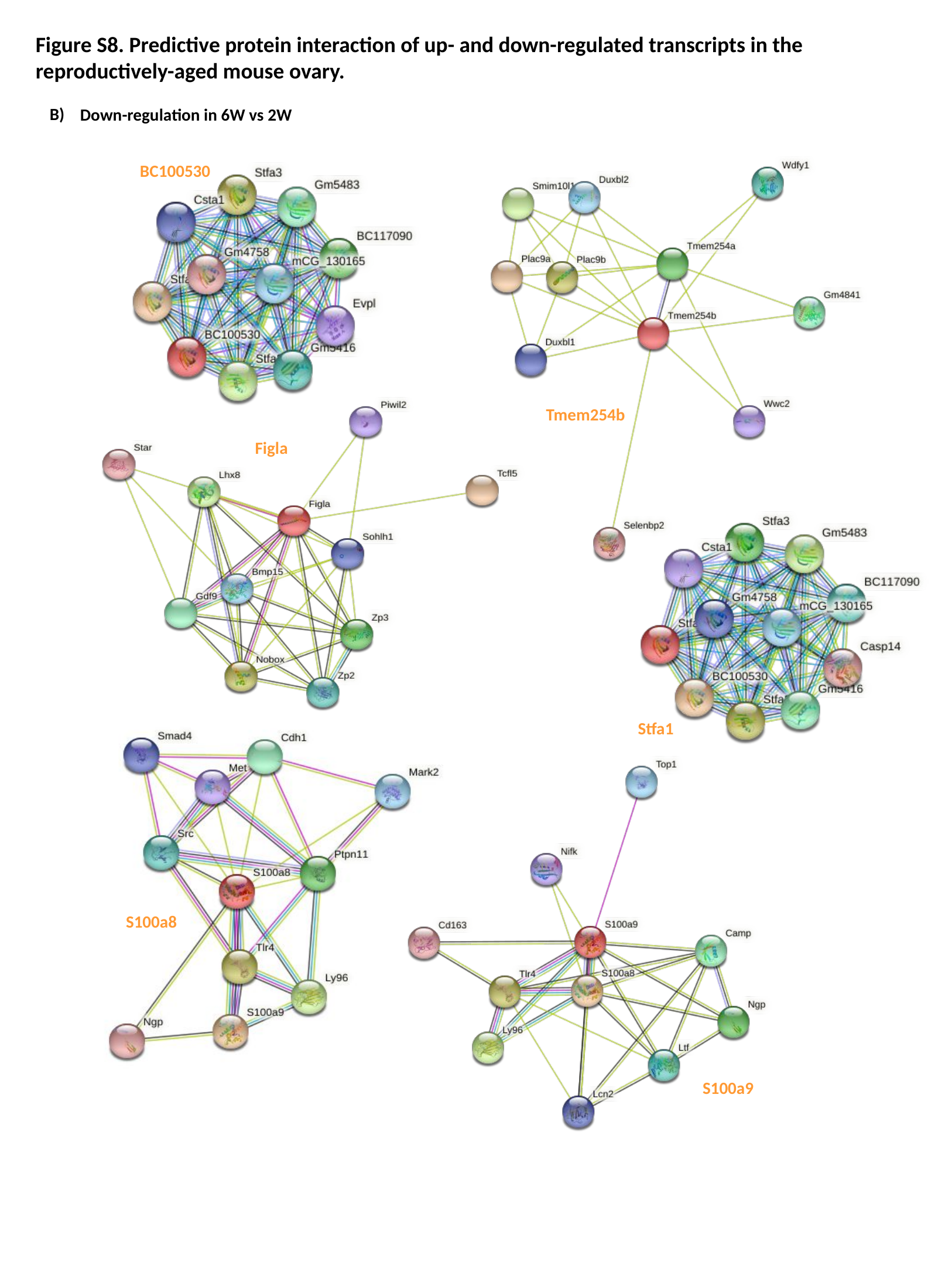

Figure S8. Predictive protein interaction of up- and down-regulated transcripts in the reproductively-aged mouse ovary.
B)
Down-regulation in 6W vs 2W
Tmem254b
BC100530
Figla
Stfa1
S100a8
S100a9

## Slide 24
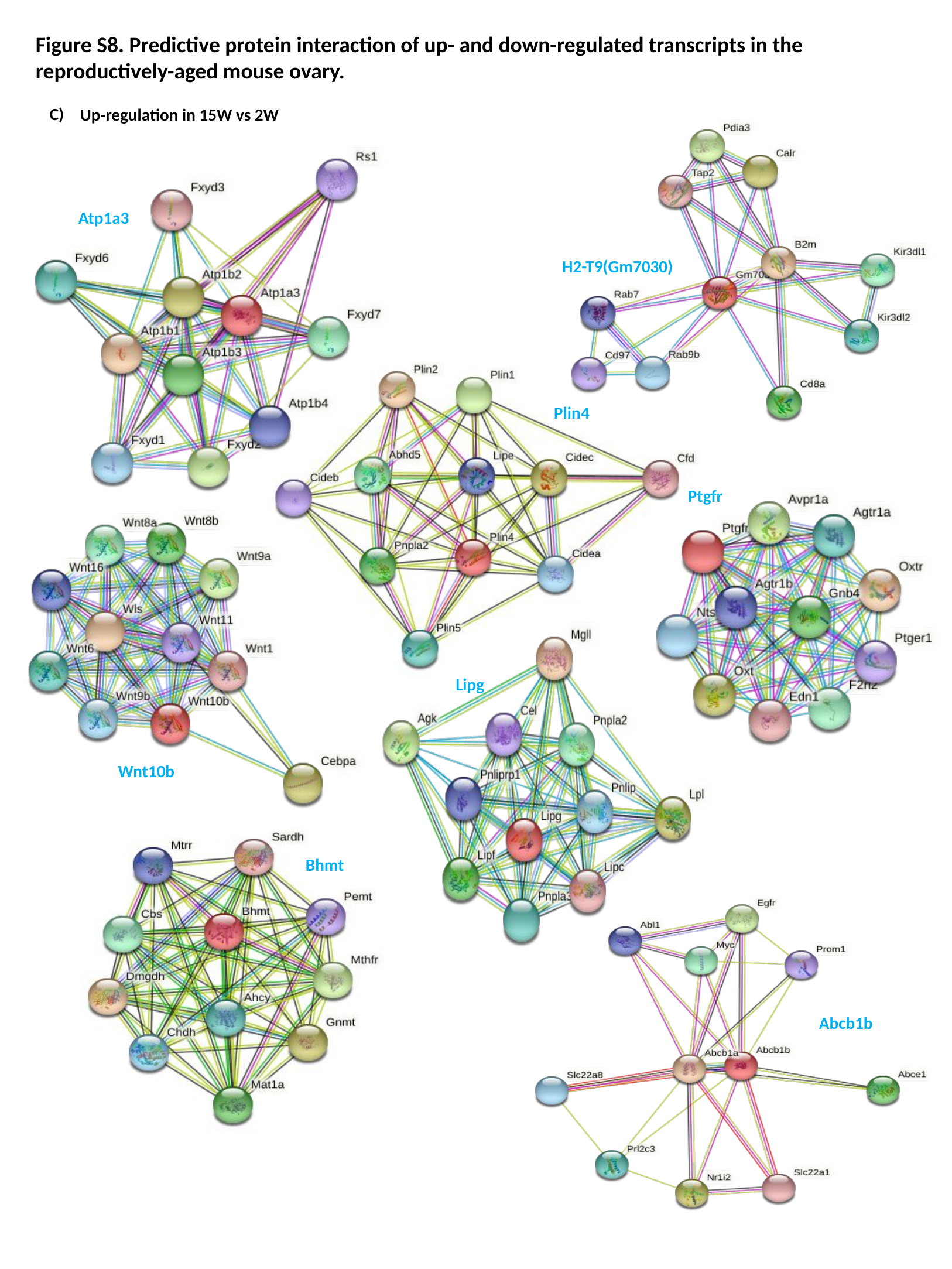

Figure S8. Predictive protein interaction of up- and down-regulated transcripts in the reproductively-aged mouse ovary.
C)
Up-regulation in 15W vs 2W
Atp1a3
H2-T9(Gm7030)
Plin4
Ptgfr
Wnt10b
Lipg
Bhmt
Abcb1b

## Slide 25
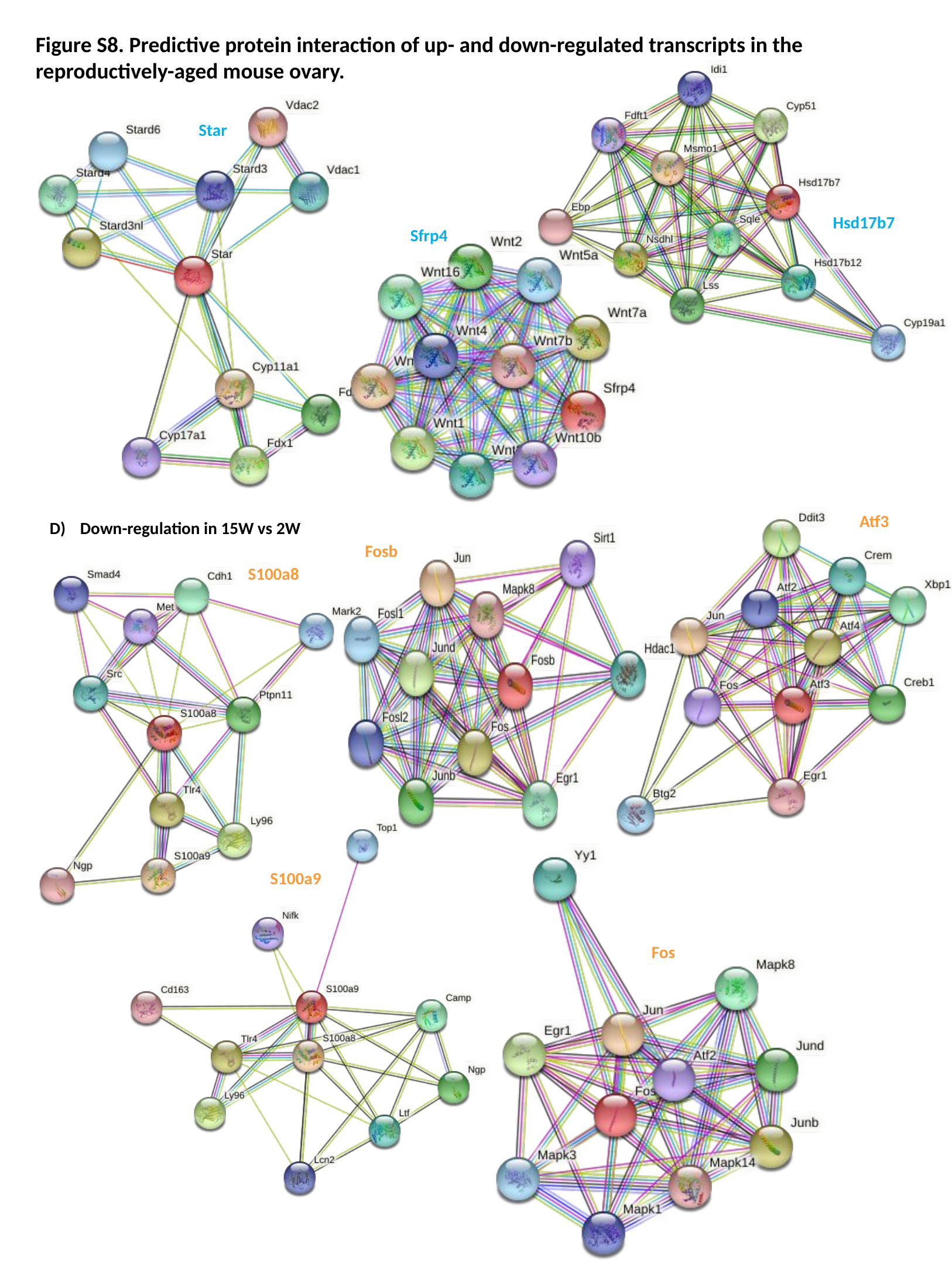

Figure S8. Predictive protein interaction of up- and down-regulated transcripts in the reproductively-aged mouse ovary.
Hsd17b7
Star
Sfrp4
Atf3
Fosb
D)
Down-regulation in 15W vs 2W
S100a8
S100a9
Fos

## Slide 26
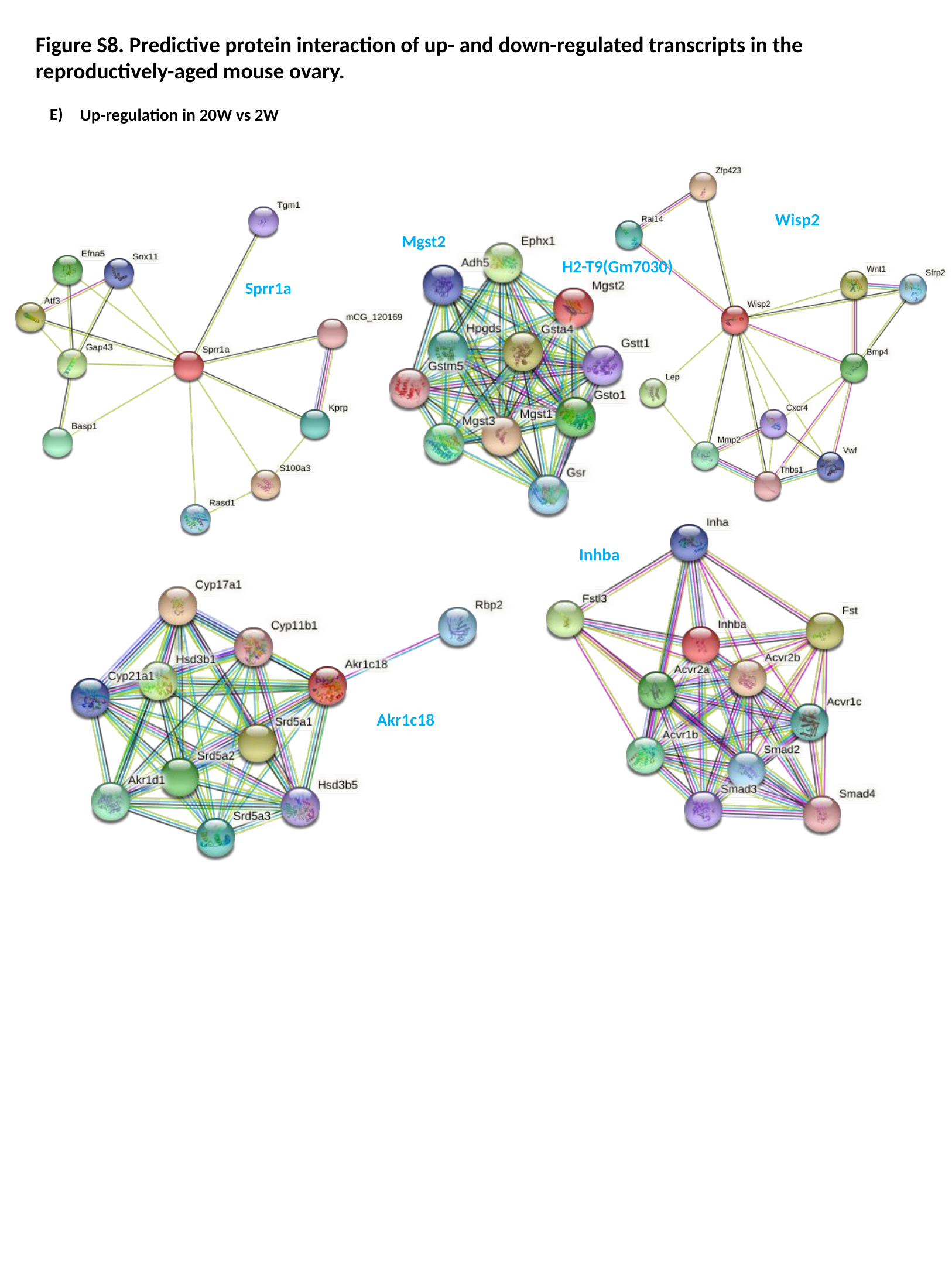

Figure S8. Predictive protein interaction of up- and down-regulated transcripts in the reproductively-aged mouse ovary.
E)
Up-regulation in 20W vs 2W
Wisp2
Sprr1a
Mgst2
H2-T9(Gm7030)
Inhba
Akr1c18

## Slide 27
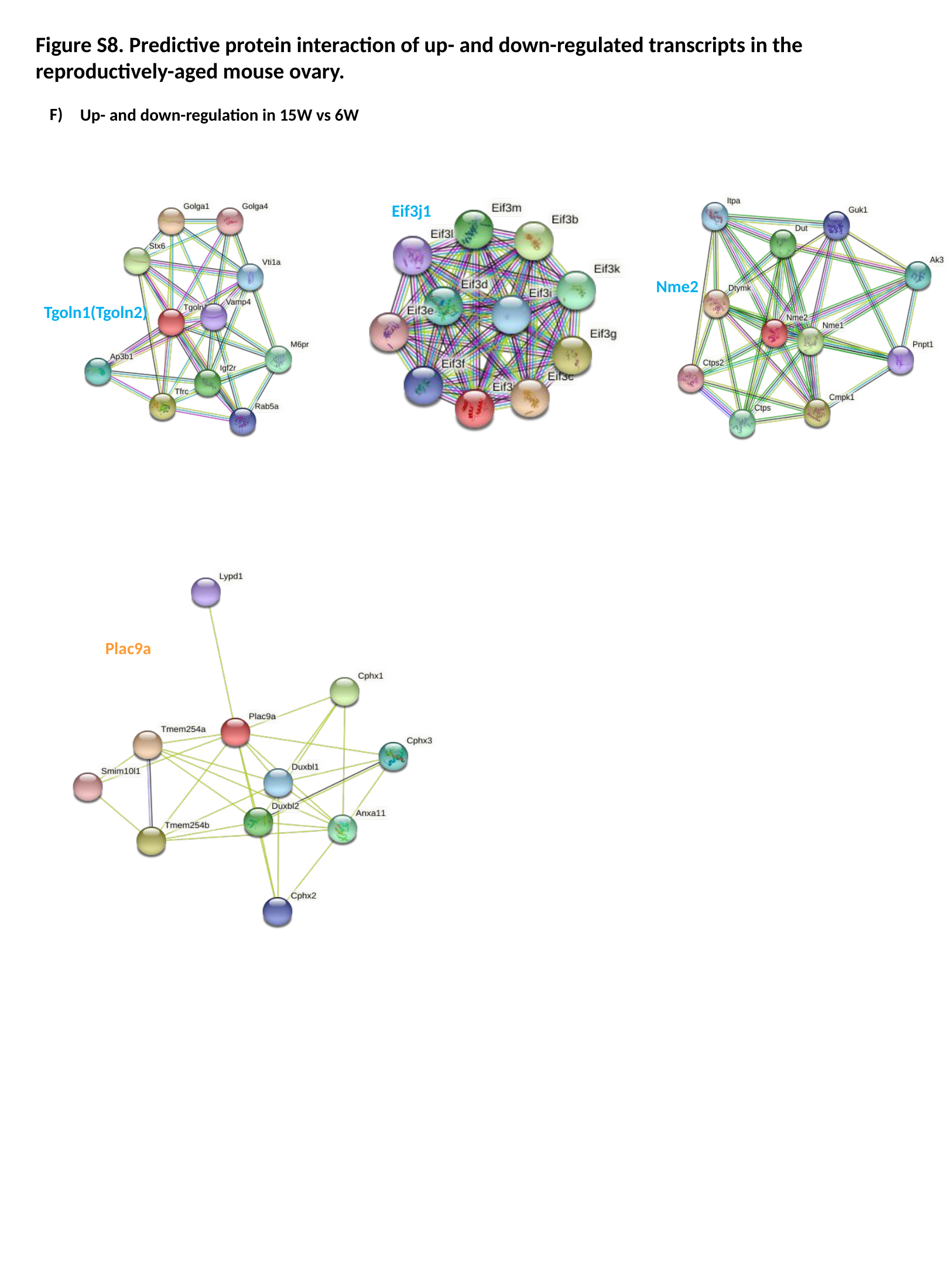

Figure S8. Predictive protein interaction of up- and down-regulated transcripts in the reproductively-aged mouse ovary.
F)
Up- and down-regulation in 15W vs 6W
Eif3j1
Nme2
Tgoln1(Tgoln2)
Plac9a

## Slide 28
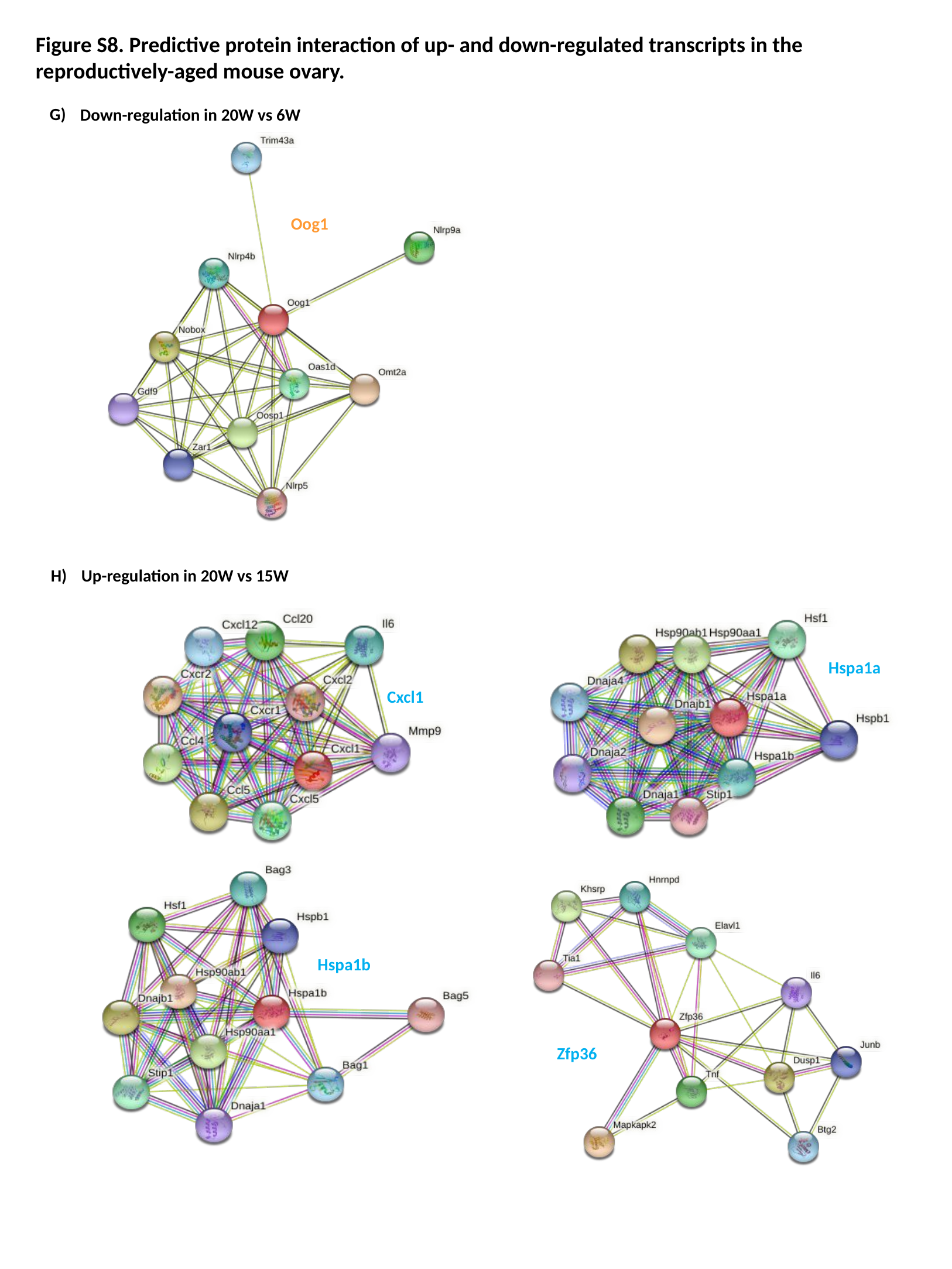

Figure S8. Predictive protein interaction of up- and down-regulated transcripts in the reproductively-aged mouse ovary.
G)
Down-regulation in 20W vs 6W
Oog1
H)
Up-regulation in 20W vs 15W
Cxcl1
Hspa1a
Hspa1b
Zfp36
