# Supplementary material for: Transcriptome Analyses Identify Potential Key microRNAs and Their Target Genes Contributing to Ovarian Reserve
Source: Int J Mol Sci. 2021 Oct 6;22(19):10819. doi: 10.3390/ijms221910819 (PMC8509654; doi:10.3390/ijms221910819)
Supplement: Supplementary file 1 [file ijms-22-10819-s001.zip › Supplementary Tables.pptx]

## Slide 1
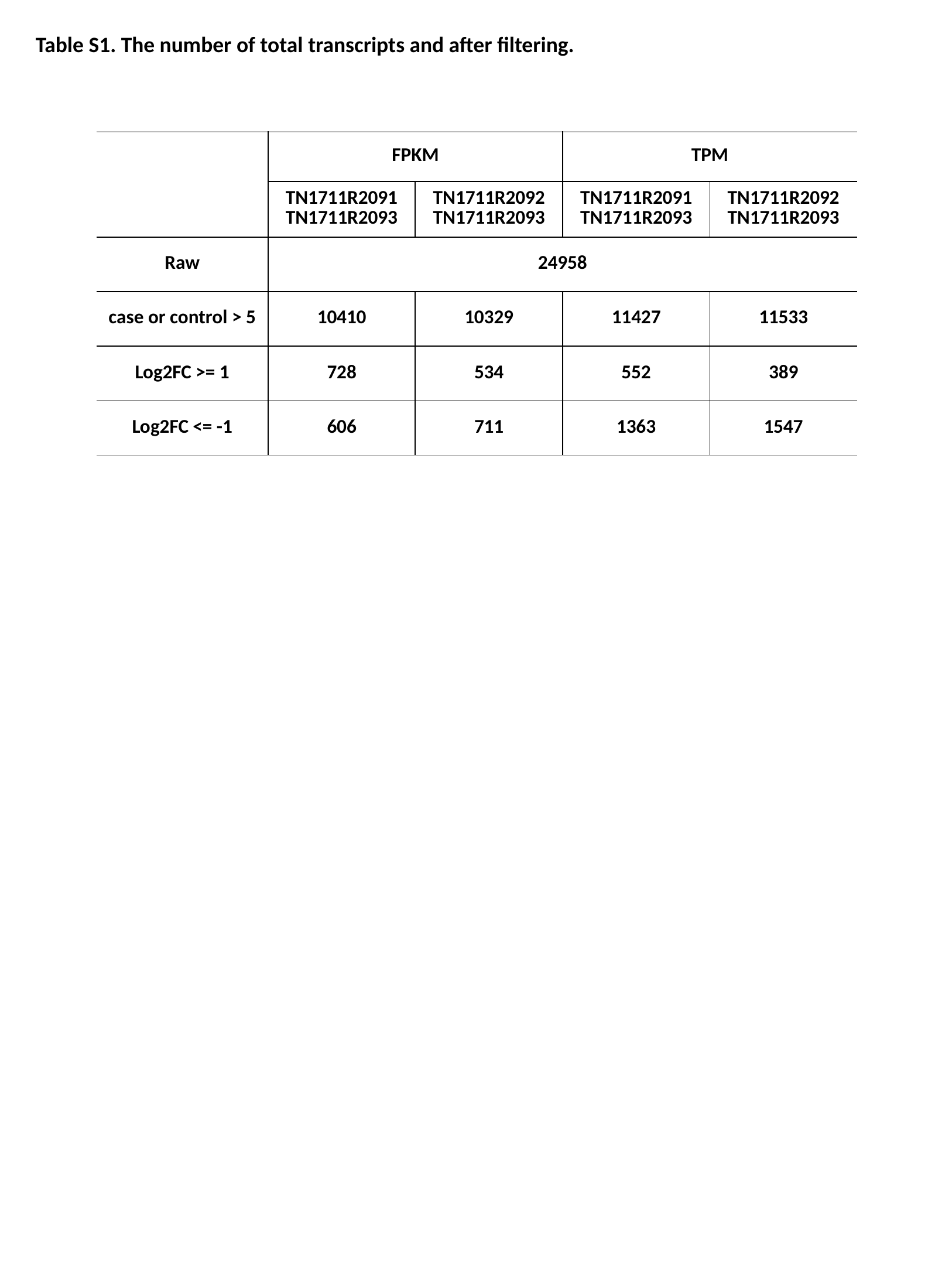

Table S1. The number of total transcripts and after filtering.
| | FPKM | | TPM | |
| --- | --- | --- | --- | --- |
| | TN1711R2091 TN1711R2093 | TN1711R2092 TN1711R2093 | TN1711R2091 TN1711R2093 | TN1711R2092 TN1711R2093 |
| Raw | 24958 | | | |
| case or control > 5 | 10410 | 10329 | 11427 | 11533 |
| Log2FC >= 1 | 728 | 534 | 552 | 389 |
| Log2FC <= -1 | 606 | 711 | 1363 | 1547 |

## Slide 2
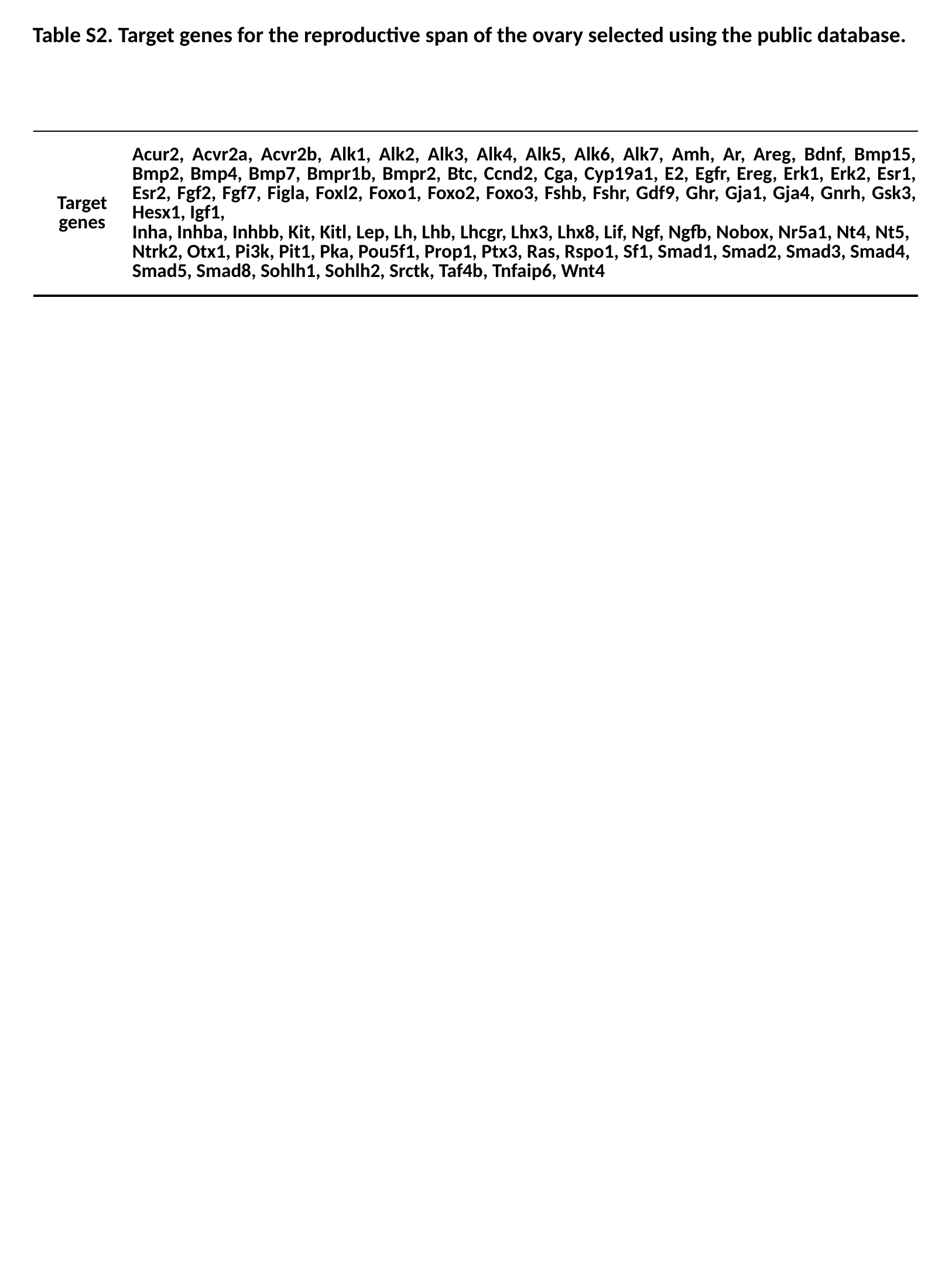

Table S2. Target genes for the reproductive span of the ovary selected using the public database.
| Target genes | Acur2, Acvr2a, Acvr2b, Alk1, Alk2, Alk3, Alk4, Alk5, Alk6, Alk7, Amh, Ar, Areg, Bdnf, Bmp15, Bmp2, Bmp4, Bmp7, Bmpr1b, Bmpr2, Btc, Ccnd2, Cga, Cyp19a1, E2, Egfr, Ereg, Erk1, Erk2, Esr1, Esr2, Fgf2, Fgf7, Figla, Foxl2, Foxo1, Foxo2, Foxo3, Fshb, Fshr, Gdf9, Ghr, Gja1, Gja4, Gnrh, Gsk3, Hesx1, Igf1, Inha, Inhba, Inhbb, Kit, Kitl, Lep, Lh, Lhb, Lhcgr, Lhx3, Lhx8, Lif, Ngf, Ngfb, Nobox, Nr5a1, Nt4, Nt5, Ntrk2, Otx1, Pi3k, Pit1, Pka, Pou5f1, Prop1, Ptx3, Ras, Rspo1, Sf1, Smad1, Smad2, Smad3, Smad4, Smad5, Smad8, Sohlh1, Sohlh2, Srctk, Taf4b, Tnfaip6, Wnt4 |
| --- | --- |

## Slide 3
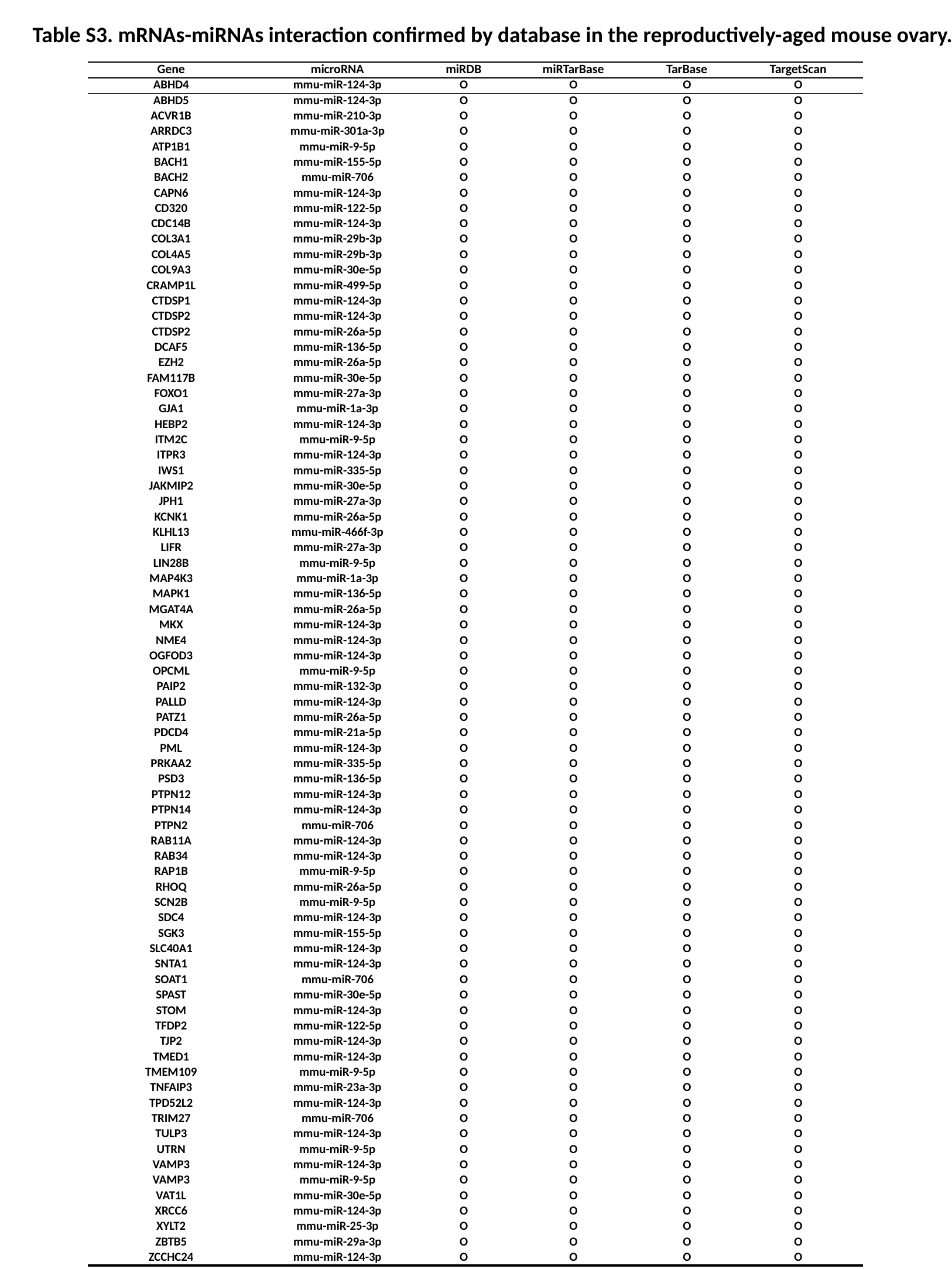

Table S3. mRNAs-miRNAs interaction confirmed by database in the reproductively-aged mouse ovary.
| Gene | microRNA | miRDB | miRTarBase | TarBase | TargetScan |
| --- | --- | --- | --- | --- | --- |
| ABHD4 | mmu-miR-124-3p | O | O | O | O |
| ABHD5 | mmu-miR-124-3p | O | O | O | O |
| ACVR1B | mmu-miR-210-3p | O | O | O | O |
| ARRDC3 | mmu-miR-301a-3p | O | O | O | O |
| ATP1B1 | mmu-miR-9-5p | O | O | O | O |
| BACH1 | mmu-miR-155-5p | O | O | O | O |
| BACH2 | mmu-miR-706 | O | O | O | O |
| CAPN6 | mmu-miR-124-3p | O | O | O | O |
| CD320 | mmu-miR-122-5p | O | O | O | O |
| CDC14B | mmu-miR-124-3p | O | O | O | O |
| COL3A1 | mmu-miR-29b-3p | O | O | O | O |
| COL4A5 | mmu-miR-29b-3p | O | O | O | O |
| COL9A3 | mmu-miR-30e-5p | O | O | O | O |
| CRAMP1L | mmu-miR-499-5p | O | O | O | O |
| CTDSP1 | mmu-miR-124-3p | O | O | O | O |
| CTDSP2 | mmu-miR-124-3p | O | O | O | O |
| CTDSP2 | mmu-miR-26a-5p | O | O | O | O |
| DCAF5 | mmu-miR-136-5p | O | O | O | O |
| EZH2 | mmu-miR-26a-5p | O | O | O | O |
| FAM117B | mmu-miR-30e-5p | O | O | O | O |
| FOXO1 | mmu-miR-27a-3p | O | O | O | O |
| GJA1 | mmu-miR-1a-3p | O | O | O | O |
| HEBP2 | mmu-miR-124-3p | O | O | O | O |
| ITM2C | mmu-miR-9-5p | O | O | O | O |
| ITPR3 | mmu-miR-124-3p | O | O | O | O |
| IWS1 | mmu-miR-335-5p | O | O | O | O |
| JAKMIP2 | mmu-miR-30e-5p | O | O | O | O |
| JPH1 | mmu-miR-27a-3p | O | O | O | O |
| KCNK1 | mmu-miR-26a-5p | O | O | O | O |
| KLHL13 | mmu-miR-466f-3p | O | O | O | O |
| LIFR | mmu-miR-27a-3p | O | O | O | O |
| LIN28B | mmu-miR-9-5p | O | O | O | O |
| MAP4K3 | mmu-miR-1a-3p | O | O | O | O |
| MAPK1 | mmu-miR-136-5p | O | O | O | O |
| MGAT4A | mmu-miR-26a-5p | O | O | O | O |
| MKX | mmu-miR-124-3p | O | O | O | O |
| NME4 | mmu-miR-124-3p | O | O | O | O |
| OGFOD3 | mmu-miR-124-3p | O | O | O | O |
| OPCML | mmu-miR-9-5p | O | O | O | O |
| PAIP2 | mmu-miR-132-3p | O | O | O | O |
| PALLD | mmu-miR-124-3p | O | O | O | O |
| PATZ1 | mmu-miR-26a-5p | O | O | O | O |
| PDCD4 | mmu-miR-21a-5p | O | O | O | O |
| PML | mmu-miR-124-3p | O | O | O | O |
| PRKAA2 | mmu-miR-335-5p | O | O | O | O |
| PSD3 | mmu-miR-136-5p | O | O | O | O |
| PTPN12 | mmu-miR-124-3p | O | O | O | O |
| PTPN14 | mmu-miR-124-3p | O | O | O | O |
| PTPN2 | mmu-miR-706 | O | O | O | O |
| RAB11A | mmu-miR-124-3p | O | O | O | O |
| RAB34 | mmu-miR-124-3p | O | O | O | O |
| RAP1B | mmu-miR-9-5p | O | O | O | O |
| RHOQ | mmu-miR-26a-5p | O | O | O | O |
| SCN2B | mmu-miR-9-5p | O | O | O | O |
| SDC4 | mmu-miR-124-3p | O | O | O | O |
| SGK3 | mmu-miR-155-5p | O | O | O | O |
| SLC40A1 | mmu-miR-124-3p | O | O | O | O |
| SNTA1 | mmu-miR-124-3p | O | O | O | O |
| SOAT1 | mmu-miR-706 | O | O | O | O |
| SPAST | mmu-miR-30e-5p | O | O | O | O |
| STOM | mmu-miR-124-3p | O | O | O | O |
| TFDP2 | mmu-miR-122-5p | O | O | O | O |
| TJP2 | mmu-miR-124-3p | O | O | O | O |
| TMED1 | mmu-miR-124-3p | O | O | O | O |
| TMEM109 | mmu-miR-9-5p | O | O | O | O |
| TNFAIP3 | mmu-miR-23a-3p | O | O | O | O |
| TPD52L2 | mmu-miR-124-3p | O | O | O | O |
| TRIM27 | mmu-miR-706 | O | O | O | O |
| TULP3 | mmu-miR-124-3p | O | O | O | O |
| UTRN | mmu-miR-9-5p | O | O | O | O |
| VAMP3 | mmu-miR-124-3p | O | O | O | O |
| VAMP3 | mmu-miR-9-5p | O | O | O | O |
| VAT1L | mmu-miR-30e-5p | O | O | O | O |
| XRCC6 | mmu-miR-124-3p | O | O | O | O |
| XYLT2 | mmu-miR-25-3p | O | O | O | O |
| ZBTB5 | mmu-miR-29a-3p | O | O | O | O |
| ZCCHC24 | mmu-miR-124-3p | O | O | O | O |

## Slide 4
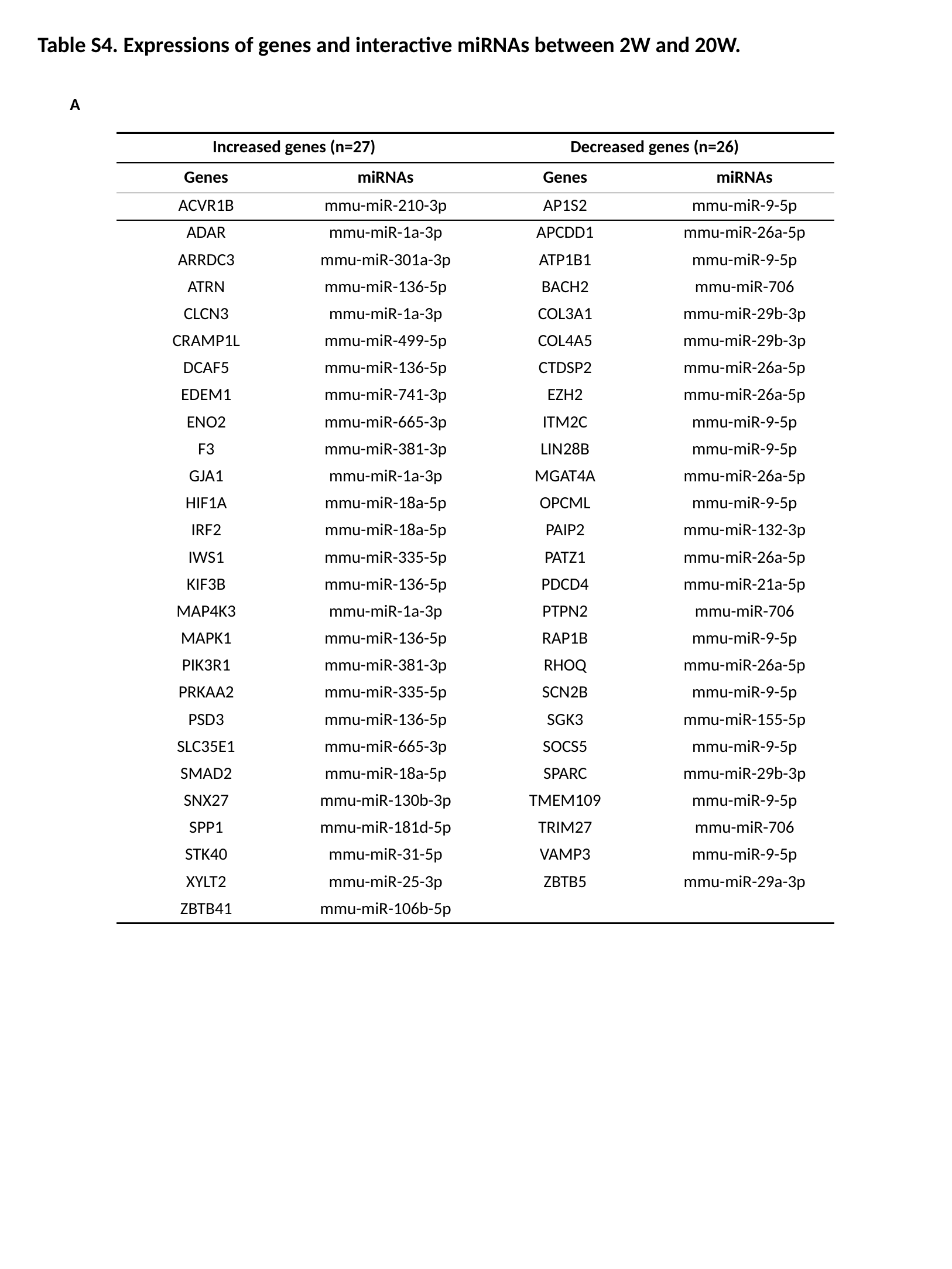

Table S4. Expressions of genes and interactive miRNAs between 2W and 20W.
A
| Increased genes (n=27) | | Decreased genes (n=26) | |
| --- | --- | --- | --- |
| Genes | miRNAs | Genes | miRNAs |
| ACVR1B | mmu-miR-210-3p | AP1S2 | mmu-miR-9-5p |
| ADAR | mmu-miR-1a-3p | APCDD1 | mmu-miR-26a-5p |
| ARRDC3 | mmu-miR-301a-3p | ATP1B1 | mmu-miR-9-5p |
| ATRN | mmu-miR-136-5p | BACH2 | mmu-miR-706 |
| CLCN3 | mmu-miR-1a-3p | COL3A1 | mmu-miR-29b-3p |
| CRAMP1L | mmu-miR-499-5p | COL4A5 | mmu-miR-29b-3p |
| DCAF5 | mmu-miR-136-5p | CTDSP2 | mmu-miR-26a-5p |
| EDEM1 | mmu-miR-741-3p | EZH2 | mmu-miR-26a-5p |
| ENO2 | mmu-miR-665-3p | ITM2C | mmu-miR-9-5p |
| F3 | mmu-miR-381-3p | LIN28B | mmu-miR-9-5p |
| GJA1 | mmu-miR-1a-3p | MGAT4A | mmu-miR-26a-5p |
| HIF1A | mmu-miR-18a-5p | OPCML | mmu-miR-9-5p |
| IRF2 | mmu-miR-18a-5p | PAIP2 | mmu-miR-132-3p |
| IWS1 | mmu-miR-335-5p | PATZ1 | mmu-miR-26a-5p |
| KIF3B | mmu-miR-136-5p | PDCD4 | mmu-miR-21a-5p |
| MAP4K3 | mmu-miR-1a-3p | PTPN2 | mmu-miR-706 |
| MAPK1 | mmu-miR-136-5p | RAP1B | mmu-miR-9-5p |
| PIK3R1 | mmu-miR-381-3p | RHOQ | mmu-miR-26a-5p |
| PRKAA2 | mmu-miR-335-5p | SCN2B | mmu-miR-9-5p |
| PSD3 | mmu-miR-136-5p | SGK3 | mmu-miR-155-5p |
| SLC35E1 | mmu-miR-665-3p | SOCS5 | mmu-miR-9-5p |
| SMAD2 | mmu-miR-18a-5p | SPARC | mmu-miR-29b-3p |
| SNX27 | mmu-miR-130b-3p | TMEM109 | mmu-miR-9-5p |
| SPP1 | mmu-miR-181d-5p | TRIM27 | mmu-miR-706 |
| STK40 | mmu-miR-31-5p | VAMP3 | mmu-miR-9-5p |
| XYLT2 | mmu-miR-25-3p | ZBTB5 | mmu-miR-29a-3p |
| ZBTB41 | mmu-miR-106b-5p | | |

## Slide 5
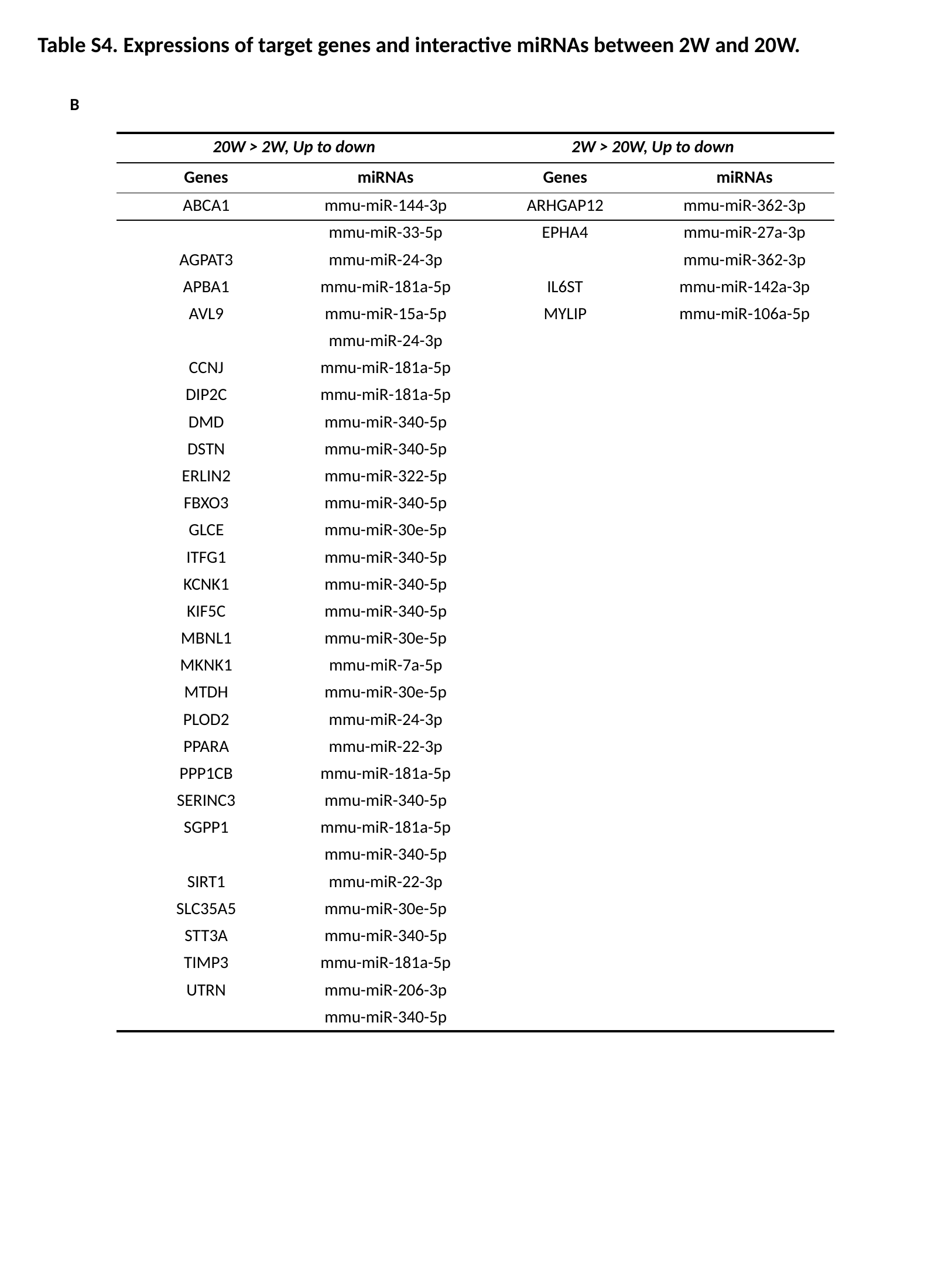

Table S4. Expressions of target genes and interactive miRNAs between 2W and 20W.
B
| 20W > 2W, Up to down | | 2W > 20W, Up to down | |
| --- | --- | --- | --- |
| Genes | miRNAs | Genes | miRNAs |
| ABCA1 | mmu-miR-144-3p | ARHGAP12 | mmu-miR-362-3p |
| | mmu-miR-33-5p | EPHA4 | mmu-miR-27a-3p |
| AGPAT3 | mmu-miR-24-3p | | mmu-miR-362-3p |
| APBA1 | mmu-miR-181a-5p | IL6ST | mmu-miR-142a-3p |
| AVL9 | mmu-miR-15a-5p | MYLIP | mmu-miR-106a-5p |
| | mmu-miR-24-3p | | |
| CCNJ | mmu-miR-181a-5p | | |
| DIP2C | mmu-miR-181a-5p | | |
| DMD | mmu-miR-340-5p | | |
| DSTN | mmu-miR-340-5p | | |
| ERLIN2 | mmu-miR-322-5p | | |
| FBXO3 | mmu-miR-340-5p | | |
| GLCE | mmu-miR-30e-5p | | |
| ITFG1 | mmu-miR-340-5p | | |
| KCNK1 | mmu-miR-340-5p | | |
| KIF5C | mmu-miR-340-5p | | |
| MBNL1 | mmu-miR-30e-5p | | |
| MKNK1 | mmu-miR-7a-5p | | |
| MTDH | mmu-miR-30e-5p | | |
| PLOD2 | mmu-miR-24-3p | | |
| PPARA | mmu-miR-22-3p | | |
| PPP1CB | mmu-miR-181a-5p | | |
| SERINC3 | mmu-miR-340-5p | | |
| SGPP1 | mmu-miR-181a-5p | | |
| | mmu-miR-340-5p | | |
| SIRT1 | mmu-miR-22-3p | | |
| SLC35A5 | mmu-miR-30e-5p | | |
| STT3A | mmu-miR-340-5p | | |
| TIMP3 | mmu-miR-181a-5p | | |
| UTRN | mmu-miR-206-3p | | |
| | mmu-miR-340-5p | | |

## Slide 6
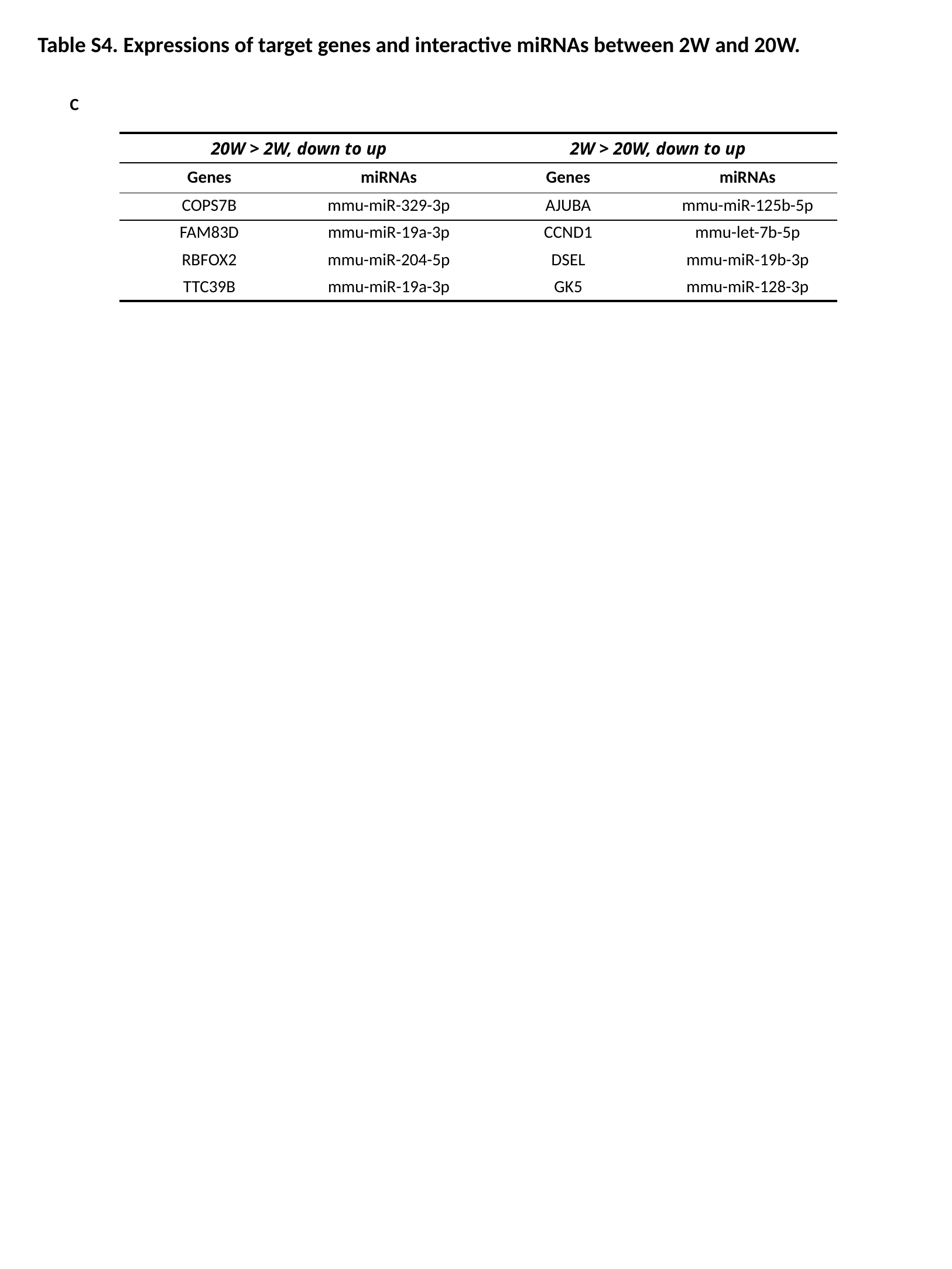

Table S4. Expressions of target genes and interactive miRNAs between 2W and 20W.
C
| 20W > 2W, down to up | | 2W > 20W, down to up | |
| --- | --- | --- | --- |
| Genes | miRNAs | Genes | miRNAs |
| COPS7B | mmu-miR-329-3p | AJUBA | mmu-miR-125b-5p |
| FAM83D | mmu-miR-19a-3p | CCND1 | mmu-let-7b-5p |
| RBFOX2 | mmu-miR-204-5p | DSEL | mmu-miR-19b-3p |
| TTC39B | mmu-miR-19a-3p | GK5 | mmu-miR-128-3p |

## Slide 7
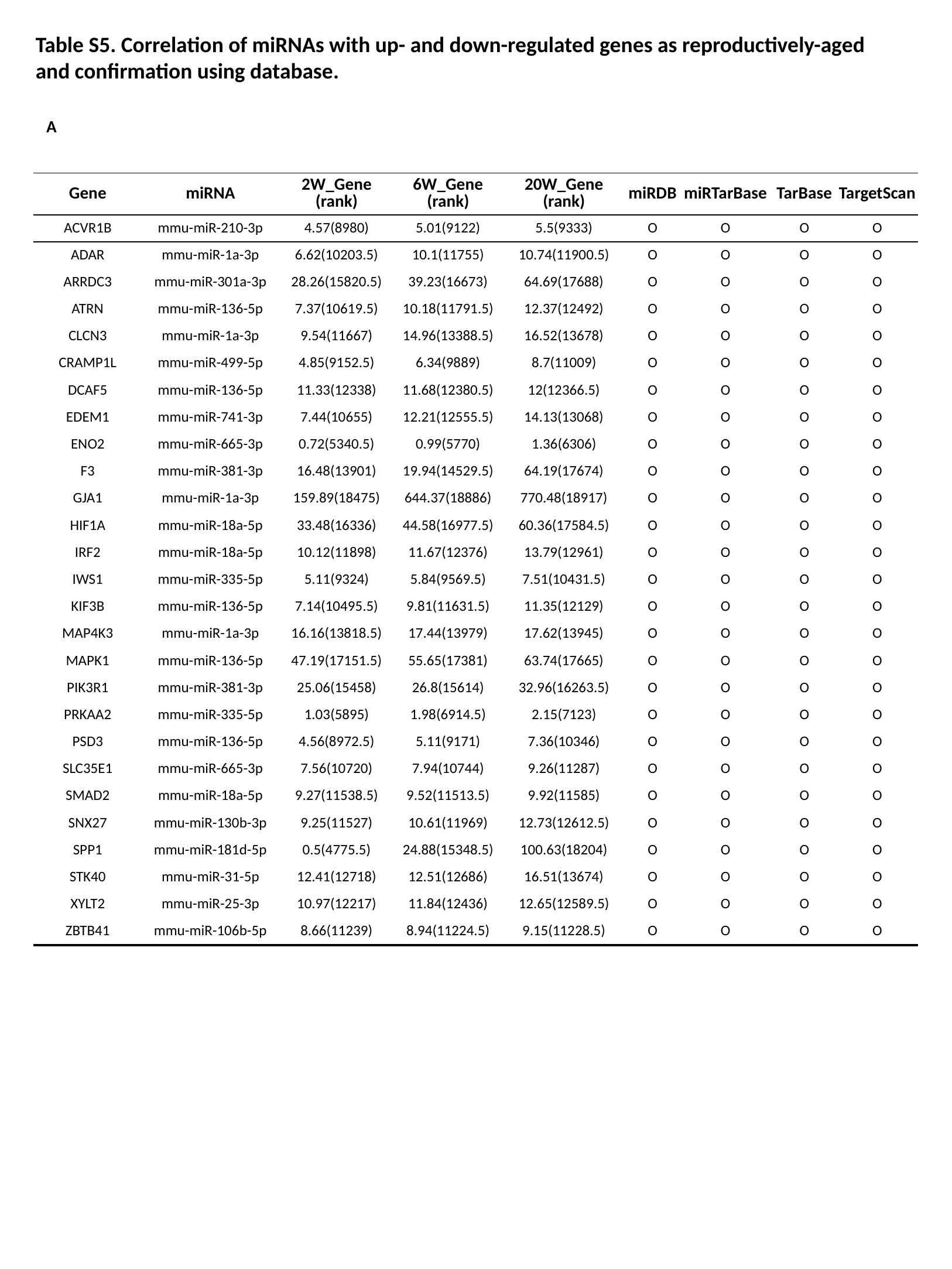

Table S5. Correlation of miRNAs with up- and down-regulated genes as reproductively-aged and confirmation using database.
A
| Gene | miRNA | 2W\_Gene (rank) | 6W\_Gene (rank) | 20W\_Gene (rank) | miRDB | miRTarBase | TarBase | TargetScan |
| --- | --- | --- | --- | --- | --- | --- | --- | --- |
| ACVR1B | mmu-miR-210-3p | 4.57(8980) | 5.01(9122) | 5.5(9333) | O | O | O | O |
| ADAR | mmu-miR-1a-3p | 6.62(10203.5) | 10.1(11755) | 10.74(11900.5) | O | O | O | O |
| ARRDC3 | mmu-miR-301a-3p | 28.26(15820.5) | 39.23(16673) | 64.69(17688) | O | O | O | O |
| ATRN | mmu-miR-136-5p | 7.37(10619.5) | 10.18(11791.5) | 12.37(12492) | O | O | O | O |
| CLCN3 | mmu-miR-1a-3p | 9.54(11667) | 14.96(13388.5) | 16.52(13678) | O | O | O | O |
| CRAMP1L | mmu-miR-499-5p | 4.85(9152.5) | 6.34(9889) | 8.7(11009) | O | O | O | O |
| DCAF5 | mmu-miR-136-5p | 11.33(12338) | 11.68(12380.5) | 12(12366.5) | O | O | O | O |
| EDEM1 | mmu-miR-741-3p | 7.44(10655) | 12.21(12555.5) | 14.13(13068) | O | O | O | O |
| ENO2 | mmu-miR-665-3p | 0.72(5340.5) | 0.99(5770) | 1.36(6306) | O | O | O | O |
| F3 | mmu-miR-381-3p | 16.48(13901) | 19.94(14529.5) | 64.19(17674) | O | O | O | O |
| GJA1 | mmu-miR-1a-3p | 159.89(18475) | 644.37(18886) | 770.48(18917) | O | O | O | O |
| HIF1A | mmu-miR-18a-5p | 33.48(16336) | 44.58(16977.5) | 60.36(17584.5) | O | O | O | O |
| IRF2 | mmu-miR-18a-5p | 10.12(11898) | 11.67(12376) | 13.79(12961) | O | O | O | O |
| IWS1 | mmu-miR-335-5p | 5.11(9324) | 5.84(9569.5) | 7.51(10431.5) | O | O | O | O |
| KIF3B | mmu-miR-136-5p | 7.14(10495.5) | 9.81(11631.5) | 11.35(12129) | O | O | O | O |
| MAP4K3 | mmu-miR-1a-3p | 16.16(13818.5) | 17.44(13979) | 17.62(13945) | O | O | O | O |
| MAPK1 | mmu-miR-136-5p | 47.19(17151.5) | 55.65(17381) | 63.74(17665) | O | O | O | O |
| PIK3R1 | mmu-miR-381-3p | 25.06(15458) | 26.8(15614) | 32.96(16263.5) | O | O | O | O |
| PRKAA2 | mmu-miR-335-5p | 1.03(5895) | 1.98(6914.5) | 2.15(7123) | O | O | O | O |
| PSD3 | mmu-miR-136-5p | 4.56(8972.5) | 5.11(9171) | 7.36(10346) | O | O | O | O |
| SLC35E1 | mmu-miR-665-3p | 7.56(10720) | 7.94(10744) | 9.26(11287) | O | O | O | O |
| SMAD2 | mmu-miR-18a-5p | 9.27(11538.5) | 9.52(11513.5) | 9.92(11585) | O | O | O | O |
| SNX27 | mmu-miR-130b-3p | 9.25(11527) | 10.61(11969) | 12.73(12612.5) | O | O | O | O |
| SPP1 | mmu-miR-181d-5p | 0.5(4775.5) | 24.88(15348.5) | 100.63(18204) | O | O | O | O |
| STK40 | mmu-miR-31-5p | 12.41(12718) | 12.51(12686) | 16.51(13674) | O | O | O | O |
| XYLT2 | mmu-miR-25-3p | 10.97(12217) | 11.84(12436) | 12.65(12589.5) | O | O | O | O |
| ZBTB41 | mmu-miR-106b-5p | 8.66(11239) | 8.94(11224.5) | 9.15(11228.5) | O | O | O | O |

## Slide 8
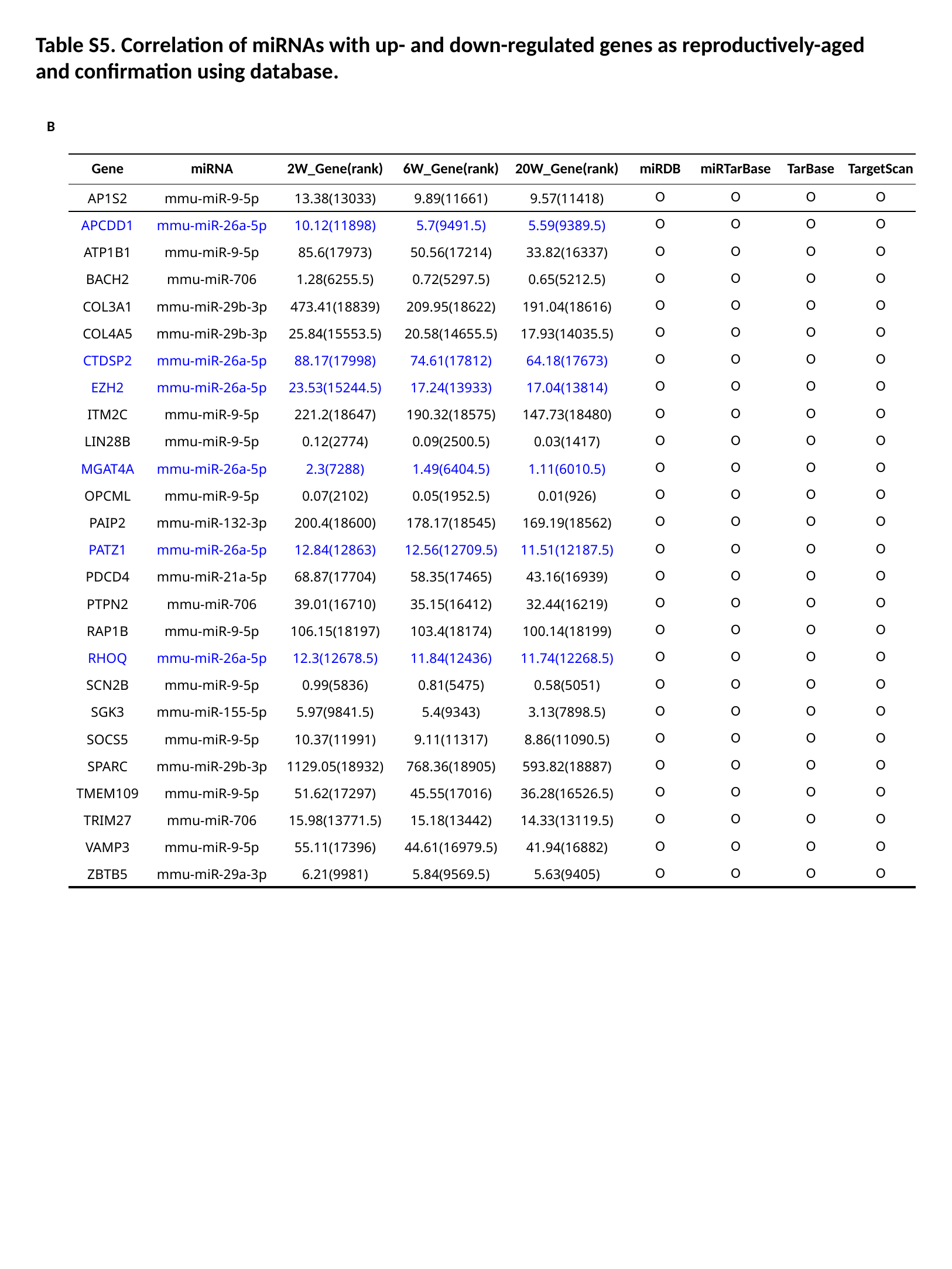

Table S5. Correlation of miRNAs with up- and down-regulated genes as reproductively-aged and confirmation using database.
B
| Gene | miRNA | 2W\_Gene(rank) | 6W\_Gene(rank) | 20W\_Gene(rank) | miRDB | miRTarBase | TarBase | TargetScan |
| --- | --- | --- | --- | --- | --- | --- | --- | --- |
| AP1S2 | mmu-miR-9-5p | 13.38(13033) | 9.89(11661) | 9.57(11418) | O | O | O | O |
| APCDD1 | mmu-miR-26a-5p | 10.12(11898) | 5.7(9491.5) | 5.59(9389.5) | O | O | O | O |
| ATP1B1 | mmu-miR-9-5p | 85.6(17973) | 50.56(17214) | 33.82(16337) | O | O | O | O |
| BACH2 | mmu-miR-706 | 1.28(6255.5) | 0.72(5297.5) | 0.65(5212.5) | O | O | O | O |
| COL3A1 | mmu-miR-29b-3p | 473.41(18839) | 209.95(18622) | 191.04(18616) | O | O | O | O |
| COL4A5 | mmu-miR-29b-3p | 25.84(15553.5) | 20.58(14655.5) | 17.93(14035.5) | O | O | O | O |
| CTDSP2 | mmu-miR-26a-5p | 88.17(17998) | 74.61(17812) | 64.18(17673) | O | O | O | O |
| EZH2 | mmu-miR-26a-5p | 23.53(15244.5) | 17.24(13933) | 17.04(13814) | O | O | O | O |
| ITM2C | mmu-miR-9-5p | 221.2(18647) | 190.32(18575) | 147.73(18480) | O | O | O | O |
| LIN28B | mmu-miR-9-5p | 0.12(2774) | 0.09(2500.5) | 0.03(1417) | O | O | O | O |
| MGAT4A | mmu-miR-26a-5p | 2.3(7288) | 1.49(6404.5) | 1.11(6010.5) | O | O | O | O |
| OPCML | mmu-miR-9-5p | 0.07(2102) | 0.05(1952.5) | 0.01(926) | O | O | O | O |
| PAIP2 | mmu-miR-132-3p | 200.4(18600) | 178.17(18545) | 169.19(18562) | O | O | O | O |
| PATZ1 | mmu-miR-26a-5p | 12.84(12863) | 12.56(12709.5) | 11.51(12187.5) | O | O | O | O |
| PDCD4 | mmu-miR-21a-5p | 68.87(17704) | 58.35(17465) | 43.16(16939) | O | O | O | O |
| PTPN2 | mmu-miR-706 | 39.01(16710) | 35.15(16412) | 32.44(16219) | O | O | O | O |
| RAP1B | mmu-miR-9-5p | 106.15(18197) | 103.4(18174) | 100.14(18199) | O | O | O | O |
| RHOQ | mmu-miR-26a-5p | 12.3(12678.5) | 11.84(12436) | 11.74(12268.5) | O | O | O | O |
| SCN2B | mmu-miR-9-5p | 0.99(5836) | 0.81(5475) | 0.58(5051) | O | O | O | O |
| SGK3 | mmu-miR-155-5p | 5.97(9841.5) | 5.4(9343) | 3.13(7898.5) | O | O | O | O |
| SOCS5 | mmu-miR-9-5p | 10.37(11991) | 9.11(11317) | 8.86(11090.5) | O | O | O | O |
| SPARC | mmu-miR-29b-3p | 1129.05(18932) | 768.36(18905) | 593.82(18887) | O | O | O | O |
| TMEM109 | mmu-miR-9-5p | 51.62(17297) | 45.55(17016) | 36.28(16526.5) | O | O | O | O |
| TRIM27 | mmu-miR-706 | 15.98(13771.5) | 15.18(13442) | 14.33(13119.5) | O | O | O | O |
| VAMP3 | mmu-miR-9-5p | 55.11(17396) | 44.61(16979.5) | 41.94(16882) | O | O | O | O |
| ZBTB5 | mmu-miR-29a-3p | 6.21(9981) | 5.84(9569.5) | 5.63(9405) | O | O | O | O |

## Slide 9
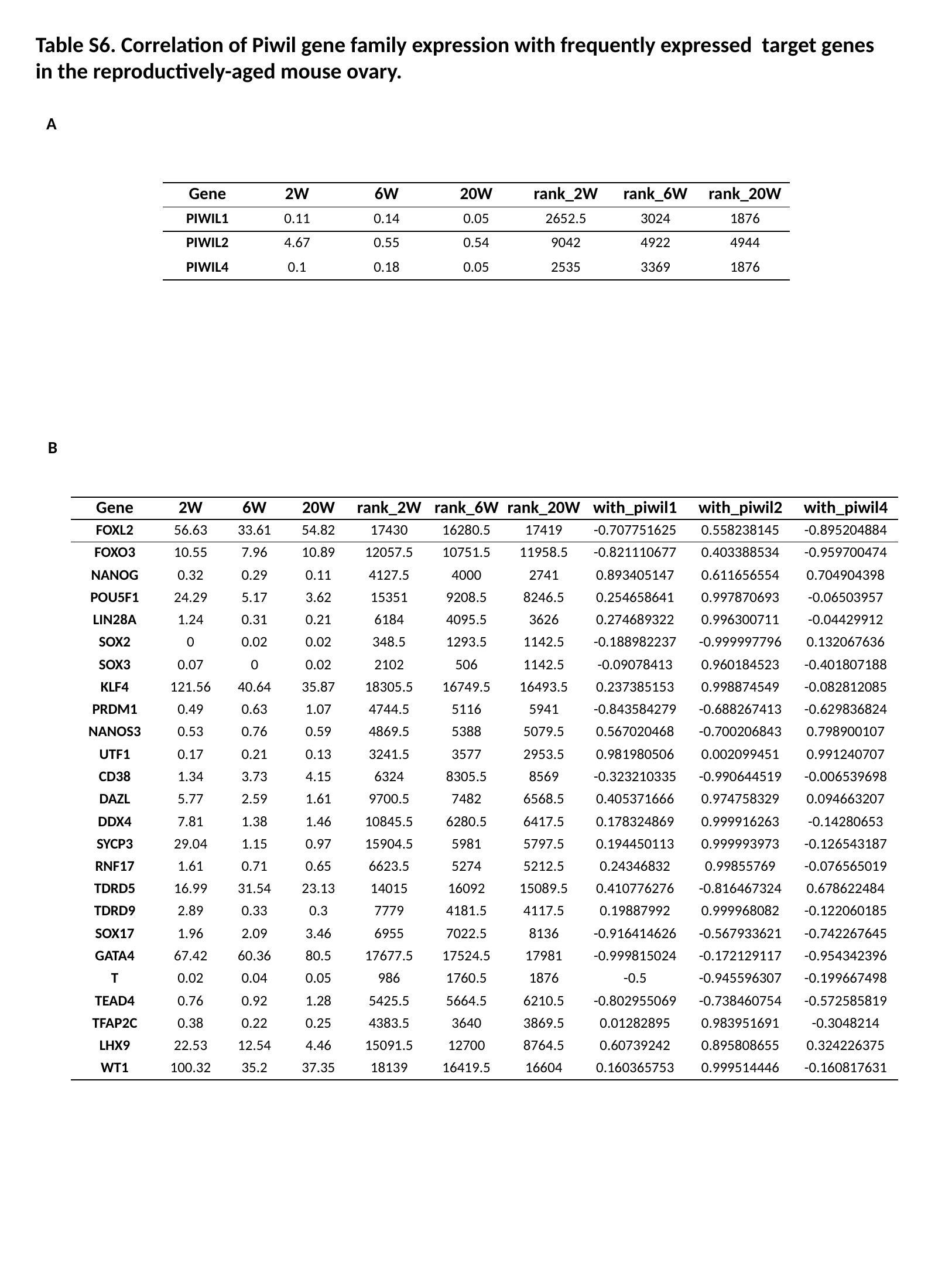

Table S6. Correlation of Piwil gene family expression with frequently expressed target genes in the reproductively-aged mouse ovary.
A
| Gene | 2W | 6W | 20W | rank\_2W | rank\_6W | rank\_20W |
| --- | --- | --- | --- | --- | --- | --- |
| PIWIL1 | 0.11 | 0.14 | 0.05 | 2652.5 | 3024 | 1876 |
| PIWIL2 | 4.67 | 0.55 | 0.54 | 9042 | 4922 | 4944 |
| PIWIL4 | 0.1 | 0.18 | 0.05 | 2535 | 3369 | 1876 |
B
| Gene | 2W | 6W | 20W | rank\_2W | rank\_6W | rank\_20W | with\_piwil1 | with\_piwil2 | with\_piwil4 |
| --- | --- | --- | --- | --- | --- | --- | --- | --- | --- |
| FOXL2 | 56.63 | 33.61 | 54.82 | 17430 | 16280.5 | 17419 | -0.707751625 | 0.558238145 | -0.895204884 |
| FOXO3 | 10.55 | 7.96 | 10.89 | 12057.5 | 10751.5 | 11958.5 | -0.821110677 | 0.403388534 | -0.959700474 |
| NANOG | 0.32 | 0.29 | 0.11 | 4127.5 | 4000 | 2741 | 0.893405147 | 0.611656554 | 0.704904398 |
| POU5F1 | 24.29 | 5.17 | 3.62 | 15351 | 9208.5 | 8246.5 | 0.254658641 | 0.997870693 | -0.06503957 |
| LIN28A | 1.24 | 0.31 | 0.21 | 6184 | 4095.5 | 3626 | 0.274689322 | 0.996300711 | -0.04429912 |
| SOX2 | 0 | 0.02 | 0.02 | 348.5 | 1293.5 | 1142.5 | -0.188982237 | -0.999997796 | 0.132067636 |
| SOX3 | 0.07 | 0 | 0.02 | 2102 | 506 | 1142.5 | -0.09078413 | 0.960184523 | -0.401807188 |
| KLF4 | 121.56 | 40.64 | 35.87 | 18305.5 | 16749.5 | 16493.5 | 0.237385153 | 0.998874549 | -0.082812085 |
| PRDM1 | 0.49 | 0.63 | 1.07 | 4744.5 | 5116 | 5941 | -0.843584279 | -0.688267413 | -0.629836824 |
| NANOS3 | 0.53 | 0.76 | 0.59 | 4869.5 | 5388 | 5079.5 | 0.567020468 | -0.700206843 | 0.798900107 |
| UTF1 | 0.17 | 0.21 | 0.13 | 3241.5 | 3577 | 2953.5 | 0.981980506 | 0.002099451 | 0.991240707 |
| CD38 | 1.34 | 3.73 | 4.15 | 6324 | 8305.5 | 8569 | -0.323210335 | -0.990644519 | -0.006539698 |
| DAZL | 5.77 | 2.59 | 1.61 | 9700.5 | 7482 | 6568.5 | 0.405371666 | 0.974758329 | 0.094663207 |
| DDX4 | 7.81 | 1.38 | 1.46 | 10845.5 | 6280.5 | 6417.5 | 0.178324869 | 0.999916263 | -0.14280653 |
| SYCP3 | 29.04 | 1.15 | 0.97 | 15904.5 | 5981 | 5797.5 | 0.194450113 | 0.999993973 | -0.126543187 |
| RNF17 | 1.61 | 0.71 | 0.65 | 6623.5 | 5274 | 5212.5 | 0.24346832 | 0.99855769 | -0.076565019 |
| TDRD5 | 16.99 | 31.54 | 23.13 | 14015 | 16092 | 15089.5 | 0.410776276 | -0.816467324 | 0.678622484 |
| TDRD9 | 2.89 | 0.33 | 0.3 | 7779 | 4181.5 | 4117.5 | 0.19887992 | 0.999968082 | -0.122060185 |
| SOX17 | 1.96 | 2.09 | 3.46 | 6955 | 7022.5 | 8136 | -0.916414626 | -0.567933621 | -0.742267645 |
| GATA4 | 67.42 | 60.36 | 80.5 | 17677.5 | 17524.5 | 17981 | -0.999815024 | -0.172129117 | -0.954342396 |
| T | 0.02 | 0.04 | 0.05 | 986 | 1760.5 | 1876 | -0.5 | -0.945596307 | -0.199667498 |
| TEAD4 | 0.76 | 0.92 | 1.28 | 5425.5 | 5664.5 | 6210.5 | -0.802955069 | -0.738460754 | -0.572585819 |
| TFAP2C | 0.38 | 0.22 | 0.25 | 4383.5 | 3640 | 3869.5 | 0.01282895 | 0.983951691 | -0.3048214 |
| LHX9 | 22.53 | 12.54 | 4.46 | 15091.5 | 12700 | 8764.5 | 0.60739242 | 0.895808655 | 0.324226375 |
| WT1 | 100.32 | 35.2 | 37.35 | 18139 | 16419.5 | 16604 | 0.160365753 | 0.999514446 | -0.160817631 |
